# Coupling CRISPR-Cas and a personal glucose meter with an enzymatic reporter for portable detection of human papillomavirus in biological samples

**DOI:** 10.7150/thno.106490

**Published:** 2025-02-03

**Authors:** Xuena Zhu, Shanshan Wang, Yuanyuan Xue, Xiaoyan Wang, Shaoqi Hu, Tingbo Liang, Wenjun Liu

**Affiliations:** 1 Department of Pathology, The First Affiliated Hospital of Zhejiang University School of Medicine, Hangzhou, 310003, China.; 2 Zhejiang Provincial Key Laboratory of Pancreatic Disease, The First Affiliated Hospital of Zhejiang University School of Medicine, Hangzhou, 310003, China.; 3 Department of Hepatobiliary and Pancreatic Surgery, The First Affiliated Hospital of Zhejiang University School of Medicine, Hangzhou, 310003, China.; 4 MOE Joint International Research Laboratory of Pancreatic Diseases, The First Affiliated Hospital of Zhejiang University School of Medicine, Hangzhou, 310003, China.; 5 The Innovation Center for the Study of Pancreatic Diseases of Zhejiang Province, Zhejiang University Cancer Center, Hangzhou, 310003, China.; 6 Cancer Center, Zhejiang University, 310058, Hangzhou, Zhejiang, China.

**Keywords:** Cas12, CRISPR, Human papillomavirus, Personal glucose meter, Invertase, POCT

## Abstract

Significant efforts and resources have been dedicated to developing CRISPR-Cas based point-of-care testing (POCT) and self-diagnosis methods for nucleic acid pathogens, aiming to complement the gold standard quantitative PCR tests, particularly in settings where centralized facilities, trained personnel, or resource-intensive equipment are unavailable. However, the reliance on stationary, high-cost readout machinery hinders their full deployment at the point of care. We aimed to develop a solid-phase invertase-labeled reporter (ILR) system that enables convenient readout of CRISPR-Cas reactions, facilitate HPV detection in a POCT-compatible manner.

**Methods:** Through multiple chemical couplings, invertase is immobilized onto magnetic microbeads via a nucleic acid linker that responds to target nucleic acid-induced CRISPR-Cas activation. This activation releases active invertase, which then converts sucrose to glucose in proportion to the target's abundance. Enzymatic signal amplification by Cas12a/Cas13a and invertase compensates for the moderate sensitivity of personal glucose meters (PGMs).

**Results:** When applied to human papillomavirus detection, the HPV18-targeted LAMP-Cas12a/ILR/PGM system can detect as few as 7 HPV18-positive HeLa cells out of 7,000, achieving 95.8% sensitivity and 100% specificity in cervical cell samples. Furthermore, minimal reagent adjustments allow for the rapid establishment of HPV16 and HPV52-targeted LAMP-Cas12a/ILR/PGM systems, offering satisfactory sensitivity, specificity, and cross-species detection.

**Conclusion:** These findings demonstrate a highly efficient testing platform for a range of nucleic acid pathogens, suitable for both point-of-care and household use.

## Introduction

The development of viral antigen, antibody, and nucleic acid-aimed testing methods prevails in viral infection screening and diagnosis. Among them, nucleic acid testing typically outperforms the other two in terms of accuracy and sensitivity, making it the most widely used diagnosis for viral infection in clinics. Nucleic acid testing is particularly advantageous for early detection of infections, such as COVID-19, where timely identification is critical, or when antibody levels are below detectable thresholds, as is the case with HPV.

Quantitative PCR compacts thermal cycler-based target amplification and fluorescence measurement into one stand-alone device and dominantly represents the gold standard in nucleic acid detection. However, its deployment is typically restricted to centralized hospitals or laboratory settings due to its large physical size, high cost, and the need for licensed operators. Consequently, there is a growing need for complementary diagnostic tests that can be implemented in low-resource settings. Such tests can be particularly crucial for managing diseases like cervical cancer, a condition characterized by persistent HPV infection. Multiple WHO-initiated studies have concluded that scale-up in coverage and frequency of pre-disease screening is effective in eliminating disease mortality 1-3.

Alternative approaches to qPCR tests for nucleic acid detection have been widely explored, with CRISPR-Cas technology emerging as a promising solution amid the global COVID-19 pandemic 4-8. The system delivered superior simplicity, as target recognition, signal transduction, and amplification can all be fulfilled by a single ribonucleoprotein (RNP) complex. Specifically, CRISPR effector proteins, including Cas12, Cas13, and Cas9, when complexed with the corresponding crRNA, exhibit trans-nuclease activity upon target binding. This process leads to the non-specific cleavage of surrounding DNA or RNA, referred to as collateral cleavage 9-11. Collateral activity against a simple fluorescence oligoribonucleotide reporter can be recorded by a fluorometer for readouts. Target specificity is governed by the spacer region of crRNA, which can be easily tailored to detect the sequence of interest.

CRISPR-Cas-based biosensing offers several advantages, including simple assay setup, rapid assay times, and isothermal reactions, making it an attractive option for the diagnosis of various pathogens 10, 12-15, genetic alterations 16, and miRNA biomarkers 17. However, target specificity remains a major concern, particularly in biological samples such as cells, blood, and urine, where interfering substances often coexist with trace amounts of target molecules. Additionally, the reliance on a fluorometer for detection, along with its high cost, impedes its widespread deployment at points of care, let alone within households.

To address these challenges, we propose integrating a personal glucose meter (PGM) with CRISPR-Cas-based testing for a POCT-compatible terminal readout. As the most widely commercialized POCT device, PGM is readily available at low cost and provides considerable accuracy (95% within ±15%, ISO 15197:2013 standard). It has already been successfully applied in biosensing development 18-22. Preceding attempts to incorporate PGM into CRISPR-Cas systems have shown promising results in various applications (Table S1) 23-31. In this study, we focused on (1) designing a more sophisticated reporter: an invertase-labeled solid-phase reporter (ILR) constructed through multiple coupling chemistries, which facilitates the conversion of nucleic acid signals into glucose; (2) integrating and validating pre-amplification, target recognition, and signal transduction components; and (3) conducting a comprehensive evaluation of diagnostic performance using both model systems and real-world cervical cell samples. Overall, our Cas/ILR/PGM system demonstrates potential as a universal biosensing platform for nucleic acid targets and has been successfully applied to HPV18, HPV16, and HPV52 DNA detection in biological samples in a self-testing compatible format.

## Results and Discussion

### Construction and validation of the invertase-labeled solid phase reporter (ILR)

**Figure 1:**
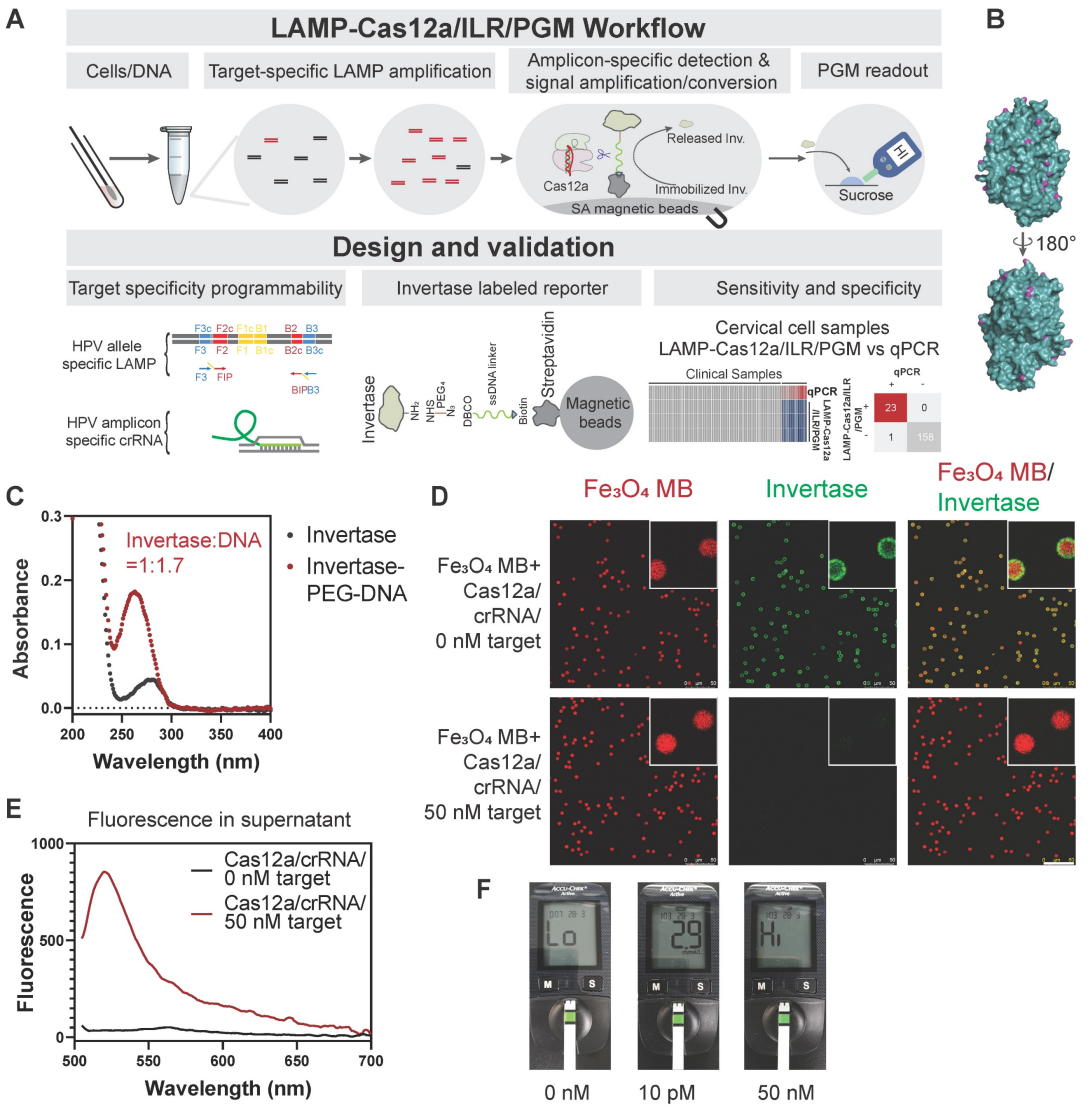
** Design and validation of the Cas12a/ILR/PGM system for DNA detection.** (A) Schematic illustration of the LAMP-Cas12a/ILR/PGM workflow and its core components. (B) Surface-located primary amines of invertase (4EQV). (C) UV-Vis absorption spectrum to reveal the composition of invertase-ssDNA conjugation. (D) Confocal fluorescence microscopy study to confirm target dependent release of invertase. Scale bar: 50 µm. (E) Fluorescence in the supernatant following invertase release. (F) PGM detection of glucose conversion catalyzed by released invertase. A model ssDNA target (miR21) was used as stimulation for D-F.

We have proposed a POCT-compatible solution for HPV detection, primarily consisting of several stages: sample lysis, pre-amplification, Cas12a/ILR reaction, and PGM readout (Figure 1A). Signal transmission from CRISPR-Cas activation to PGM readout is mediated by an enzymatically (invertase) labeled magnetic microbead reporter, constructed with a short single-stranded DNA (T_30_) oligonucleotide and a series of chemical linkages (Figure 1A). Upon activation, Cas12a induces trans-cleavage of the ssDNA linker, releasing invertase from the solid phase. Free invertase is then transferred to the PGM readout zone, where it catalyzes the conversion of sucrose to glucose, which can be quantified conveniently by a PGM. We hypothesize that the additional signal amplification provided by invertase compensates for the relatively high limit of detection (LOD) of commercial PGMs, enabling this POCT-compatible system to achieve sensitivity comparable to conventional fluorometer-based CRISPR-Cas biosensing.

Invertase possesses multiple surface-exposed primary amines (~20), making it highly suitable for chemical coupling (Figure 1B). Although all primary amines were saturated with an NHS-PEG4-N3 linker, the DNA/invertase ratio was kept low at 1.7:1 (Figure 1C and Figure S1A) to prevent the adverse effects of a higher DNA-to-enzyme ratio. A higher ratio would otherwise necessitate additional Cas12 cleavage per enzyme, thereby reducing the efficiency of enzyme release. The ILR exhibited red autofluorescence from its Fe3O4 core, which was surrounded by green fluorescence from the invertase staining under confocal fluorescence microscopy (Figure 1D, upper panels), confirming successful conjugation of the ILR reporter. Subsequently, we purified LbCas12a in-house (Figure S2A-B), ensuring it retained sufficient collateral cleavage activity when forming a ribonucleoprotein (RNP) complex with a model ssDNA target of miR-21 derived sequence and its corresponding crRNA (see Table S2 for sequence details). Cas12a activation triggered efficient invertase release from the microbeads, as indicated by the disappearance of green staining from the microbead surface (Figure 1D, lower panels) and the accumulation of green fluorescence in the supernatant (Figure 1E). These results confirmed the functionality of the Cas12a/ILR component. Further validation and optimization involved assessing the reaction times for Cas12a trans-cleavage and glucose conversion, as well as the sucrose concentration (Figure S3A-C).

To evaluate the feasibility of nucleic acid detection using the integrated Cas12a/ILR/PGM system, we observed differential PGM readouts produced by 0, 10, and 50,000 pM ssDNA targets in solution (Figure 1F). The 50 nM target resulted in a "HI" readout, exceeding the report range of PGM (0.6-33.3 mmol/L), while the 10 pM target yielded a 2.9 mmol/L readout, significantly distinct from the blank. Although a DNA/invertase ratio of 1.7:1 was initially chosen by rationale, we experimentally tested the effect of varying this ratio on the performance of the Cas12a/ILR/PGM system when stimulated by a 10 pM ssDNA target (Figure S1B). The 1.7:1 ratio produced the most efficient signal transduction and was thus used in all subsequent experiments. Additionally, a time-course analysis of ILR stability and functionality following prolonged storage suggested that the ILR could be stored at -30 °C for over 9 weeks without significant degradation or loss of activity (Figure S4A-B).

### The Cas12a/ILR/PGM POCT-compatible system retains comparable detection sensitivity as a laboratory fluorometer based assay

**Figure 2:**
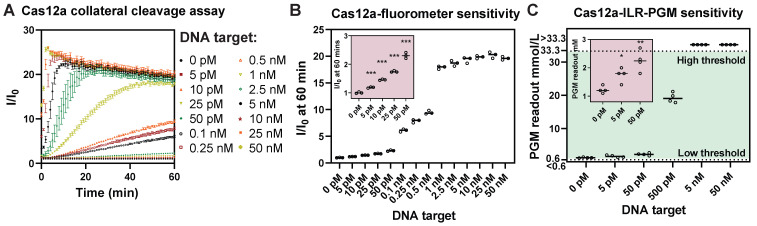
** ILR/PGM integration provides sensitivity comparable to that of a laboratory fluorometer.** (A) A typical Cas12a collateral cleavage assay. (B) LOD determination suggested an experimental LOD of 5 pM. (C) PGM readouts of the proposed Cas12a/ILR/PGM system in response to serial dilution of DNA target, showing a comparable LOD. Data points above the high threshold and below the low threshold of the PGM range correspond to values greater than 33.3 mmol/L and less than 0.6 mmol/L, respectively.

To evaluate the LOD of the Cas12a/ILR/PGM system, we compared it with a standard Cas12a/fluorometer collateral cleavage assay which uses a FAM-T_12_-BHQ reporter and a BioTek Synergy Neo2 fluorometer capable of time-course recording (Figure 2A). Analysis of the relative fluorescence signal (I/I0) at 60 min in the fluorometer based assay revealed an experimental LOD of 5 pM ssDNA target (Figure 2B). On the other hand, the Cas12a/ILR/PGM system was also able to detect as low as 5 pM of target in a similar serial dilution (Figure 2C). These results manifested that the integration of a convenient PGM device can provide satisfactory sensitivity when coupled with enzymatic (invertase) signal amplification. It is worth noting that the Accu-Chek meter, along with other commercial blood glucose meters, offers sensitivity only at the mmol/L level. We believe that further improvements in LOD of the Cas12a/ILR/PGM system by several orders of magnitude could be readily achieved through the use of a customized glucose meter module.

In addition to Cas12a, we also investigated the compatibility of Cas13a with ILR/PGM-mediated signal conversion and readout. First, the ssRNA (U_30_) linker was efficiently cleaved upon Cas13a activation (Figure S5A). Second, the Cas13a/ILR/PGM system exhibited superior sensitivity (LOD = 2.5 pM) compared to the laboratory fluorometer counterpart (LOD = 5 pM) (Figure S5B-D). These results indicate that the ILR/PGM-integrated POCT-compatible system is an effective approach for the detection of both DNA and RNA. Later in this article, we will further demonstrate the feasibility of this novel system for target-specific applications in real-world samples.

### Selection of LAMP primers for the HPV18 genome with target specificity and low self-amplification

Cervical cancer, characterized by persistent high-risk HPV infection, is the fourth most common cancer in women, causing approximately 350,000 deaths annually 32, 33. Pre-disease screening, primarily through DNA testing, has been shown to significantly reduce both cervical cancer-related mortality and the financial burden on healthcare systems 1-3. The Cas12a/ILR/PGM system was employed to develop novel POCT-compatible tests for HPV detection that can be delivered directly to end-users.

**Figure 3:**
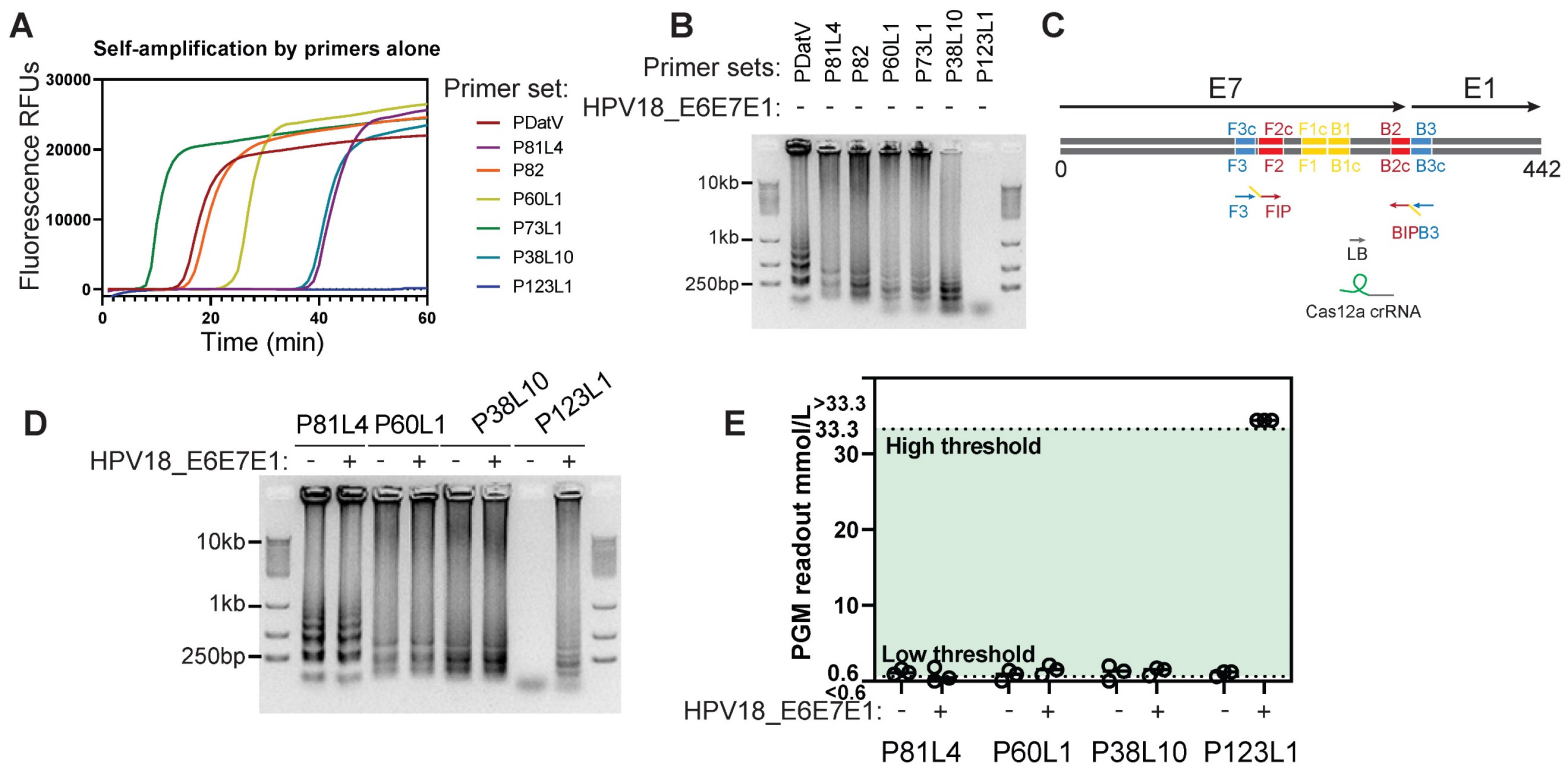
** Screening for low self-amplification LAMP primer sets of HPV18.** (A) Real-time fluorescent measurements of LAMP reactions using various primer sets alone. (B) Agarose gel showing the LAMP products from A. (C) P123L1 primers and corresponding Cas12a crRNA binding locations on the HPV18 genome. (D) Agarose gel showing products of LAMP with various primer sets and the HPV18 E6E7E1 gene template. (E) Subsequent Cas12a/ILR/PGM responses to LAMP products from D.

Due to low HPV18 abundances in cervical swab samples, direct sensing of nucleic acids in cell lysates proved impractical, as evidenced by a low signal-to-noise ratio (data not shown). Therefore, loop-mediated isothermal amplification (LAMP) was introduced for target pre-amplification. However, target-independent and primer-initiated self-amplification has been observed for numerous reported primer sets, severely compromising the reliability of LAMP-assisted biosensing 34. Among several LAMP primer sets (see Table S3 for sequence details) reported in previous studies (PDatV) or designed using the NEB LAMP primer toolset, only one primer set, P123L1, demonstrated negligible self-amplification (Figure 3A-B). This primer set targeted the boundary of the E7 and E1 genes (Figure 3C) and yielded distinctly large amplicons following template (HPV18 E6E7E1 plasmid) mediated amplification (Figure 3D). The other three primer sets, although they also exhibited relatively low levels of self-amplification in Figure 3B, conveyed undistinguishable amplification patterns between self-amplification and amplification with template presence. Further examination of these large amplicon products using Cas12a/ILR/PGM revealed that only the P123L1-mediated, template-dependent LAMP product produced a pronounced PGM response (Figure 3E), and it was selected for all subsequent experiments. Apparently, unspecific LAMP amplification could be mitigated by the specificity of Cas12a crRNA recognition.

### Detection of HPV18 DNA in solution and cellular context

**Figure 4:**
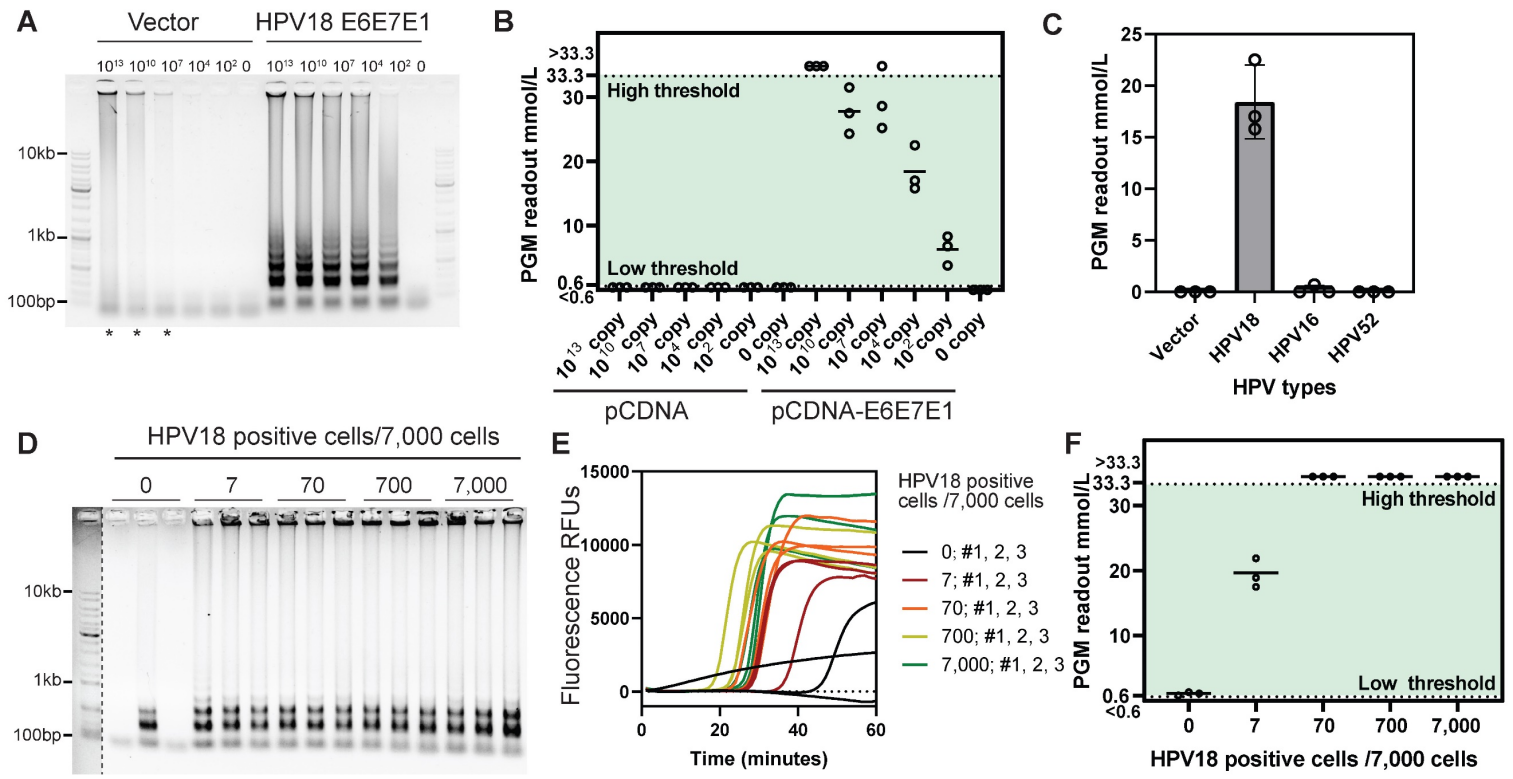
** Figure 4. Cas12a/ILR/PGM based detection of HPV18 gene in solution and cell lysates.** (A) Agarose gel showing the products of LAMP reactions with plasmid templates. (B) Subsequent Cas12a/ILR/PGM readouts of the LAMP products from A. (C) Specificity assessment of the HPV18 LAMP-Cas12a/ILR/PGM system with 10,000 copies of HPV plasmids. (D and E) Agarose gel and real-time fluorescence showing the LAMP reactions with lysates of cell mixtures containing various amounts of HeLa cells. The marker to the left of the dashed line was enhanced for clear visualization. (F) Subsequent Cas12a/ILR/PGM readouts of the LAMP products from D and E.

The pCDNA3.1 plasmid carrying HPV18 E6, E7, and E1 gene inserts was used to evaluate the performance of the Cas12a/ILR/PGM system in homogeneous solutions. LAMP alone exhibited HPV18 gene-dependent amplification, although non-specific amplification occurred with the high-concentration control plasmid (Figure 4A and Figure S6A-B). Subsequent Cas12a/ILR/PGM detection successfully distinguished 100-copy target plasmids from the blank solution with statistical significance, while refraining from non-specific amplicons (Figure 4B). Next, the full LAMP-Cas12a/ILR/PGM procedure was subjected to a specificity assessment. Among the empty vector and three HPV types tested, the system exclusively responded to the 10,000-copy HPV18 plasmid, confirming satisfactory specificity in solution (Figure 4C). Consistent with the known HPV infection status of laboratory-cultured cell lines 35, the immortal cervical epithelial cell line HeLa, but not MIA PaCa-2 or HEK-293 cells, tested positive using the Cas12a/ILR/PGM system (Figure S7A-F).

To assess the detection limit of the system, we used a series of HeLa cell mixtures with the HPV-negative cell line HEK-293. The LAMP reaction with the P123L1 primer set resulted in evident amplicons for cellular samples containing ≥ 7 HeLa cells (Figure 4D-E). Although LAMP amplification curves showed some variation within groups, an acknowledged reproducibility issue for this technology, LAMP proved effective for efficient amplification within a certain time frame. Subsequent Cas12a/ILR-PGM detection successfully discriminated the 7 HeLa cell mixture from the non-HeLa containing mixture (Figure 4F). Notably, this procedure remained inert to non-specific amplicons from the non-HeLa mixture, highlighting the system's ability to minimize false positives. Furthermore, in a head-to-head comparison with previously reported HPV detection approaches that combine pre-amplification and Cas12a trans-cleavage, we replicated the tandem RPA-Cas12a/fluorometer method (DETECTR) using optimal primers targeting the same HPV18 loci (Figure S8A). Our results demonstrated that the LAMP-Cas12a/ILR/PGM system offers higher sensitivity, detecting 7 HeLa cells out of 7,000, compared to 70 cells detected by the DETECTR approach (Figure S8B).

### Detection of HPV18 in clinical samples using the LAMP-Cas12a/ILR/PGM procedure

In 2018, WHO launched the "Cervical Cancer Elimination" initiative, with the goal of reducing cervical cancer incidence to fewer than four cases per 100,000 women by 2030 36. Scaling up the coverage and frequency of cervical cancer screening represents the top priority in the pursuit of “elimination”, particularly in low- and middle-income countries 1-3. Novel diagnostic methods with potential for wider distribution, reaching low-resource districts, communities, and even households are essential for enhancing the test coverage and frequency.

**Figure 5:**
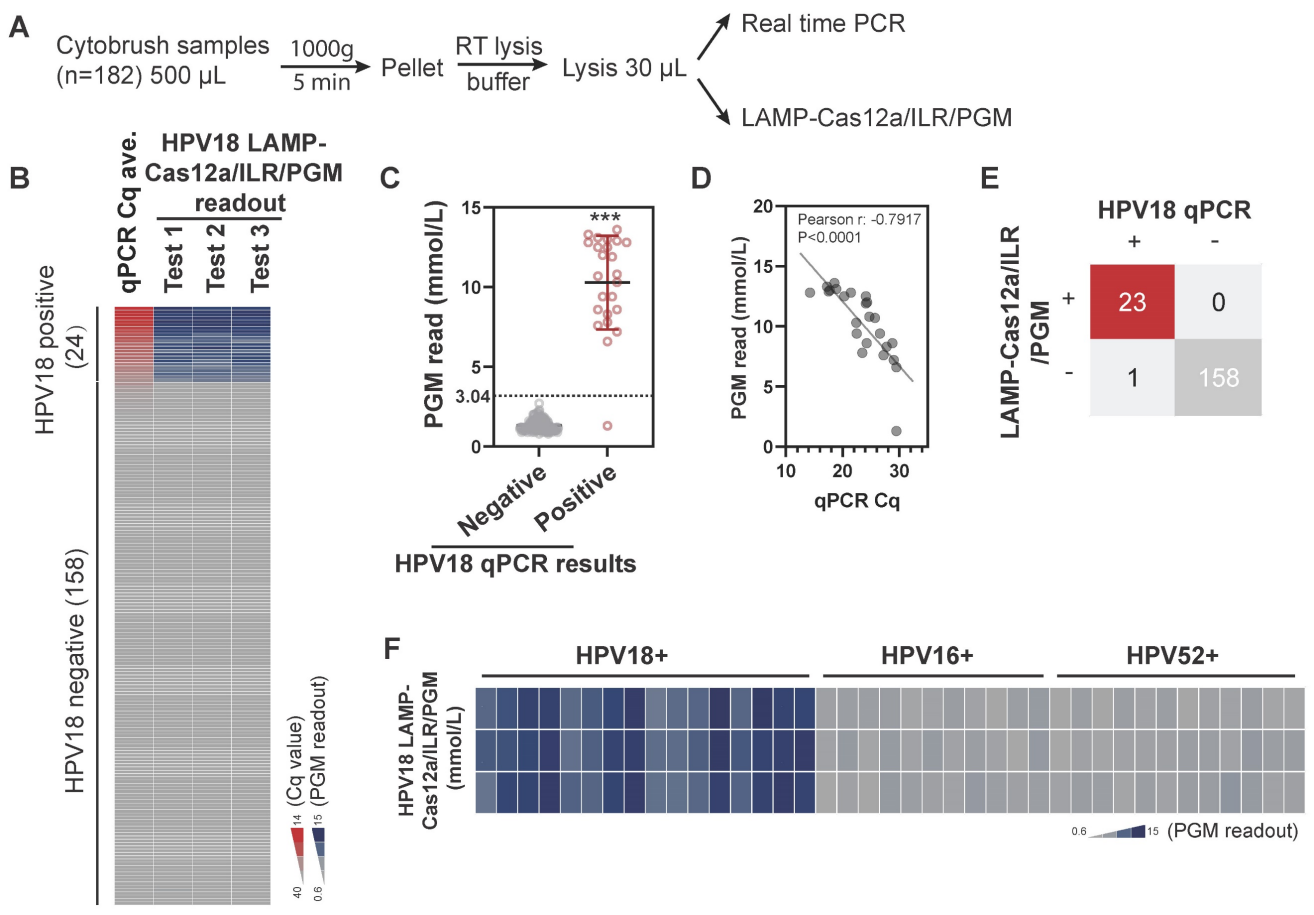
** Detection of HPV18 using the LAMP-Cas12a/ILR/PGM procedure in biological samples.** (A) Procedure for the comparative assessment. (B) Heatmap view of qPCR and the LAMP-Cas12a/ILR/PGM testing values. Samples are vertically ordered by their qPCR Cq values. (C) PGM readout comparison between the qPCR negative and positive groups. Means and SDs are indicated as solid lines. Mean+4SD value of the qPCR negative group was adopted as the threshold for the LAMP-Cas12a/ILR/PGM detection. (D) Correlation between the qPCR and PGM readouts in the qPCR positive group. (E) Concordance tables between HPV18 qPCR and the LAMP-Cas12a/ILR/PGM detections are shown for clinical samples. (F) Heatmap view of HPV18 LAMP-Cas12a/ILR/PGM detection results across a panel of HPV18, HPV16, and HPV52 positive samples.

When combined with self-collection swabs and lysis buffers designed for nucleic acid extraction at ambient temperatures, this system offers considerable potential for household-based self-testing. To assess its feasibility for cervical cell specimens, cervical cytobrush samples with existing HPV determinations were collected from an institutional hospital (n=182) and analyzed in parallel using both real-time PCR and LAMP-Cas12a/ILR/PGM methods (Figure 5A). Although the original cytobrush samples contained notable amounts of glucose, ranging from 0.1 mM to 4 mM, these levels significantly decreased to negligible concentrations (below 0.02 mM) after the LAMP-Cas12a/ILR/PGM procedure (Figure S9A-B). Therefore, the method is compatible with cytobrush samples.

For the semi-quantitative nature of the assay, we focused primarily on test sensitivity and specificity. The results from self-conducted real-time PCR were largely consistent with reported HPV18 outcomes, with all positive samples (n=24) showing Cq values below 30. The LAMP-Cas12a/ILR/PGM readouts for positive cases were significantly higher than those for negative cases (Figure 5B-C; see Table S4 for complete test results). Additionally, Cq values and PGM readouts were closely correlated within the positive group (Figure 5D), indicating a dose-dependent response of the LAMP-Cas12a/ILR/PGM system in cervical samples. The mean + 4SD value of the negative group was adopted as the threshold for LAMP-Cas12a/ILR/PGM detection. Of the 24 positive samples, 23 were correctly identified as positive, and all 158 negative samples were correctly identified as negative, resulting in a detection sensitivity of 95.8% and a specificity of 100% (Figure 5E). The relative standard deviation (RSD) for a pair of randomly selected negative and positive samples was 14.9% and 11.8%, respectively, indicating satisfactory reproducibility (Figure S10A). The positive sample was further tested using reagents prepared in their ready-to-use format and stored at -30°C for various durations, to simulate practical on-site use. The reagents exhibited optimal stability for up to two weeks (Figure S10B), providing a sufficient turnaround time for the delivery and detection of HPV18 on-site. Furthermore, the current system successfully distinguished HPV18 from other HPV strains in a panel of single HPV18, HPV16, or HPV52 positive samples (Figure 5F).

### Detection of HPV16 and HPV52 in clinical samples using the LAMP-Cas12a/ILR/PGM procedure

**Figure 6:**
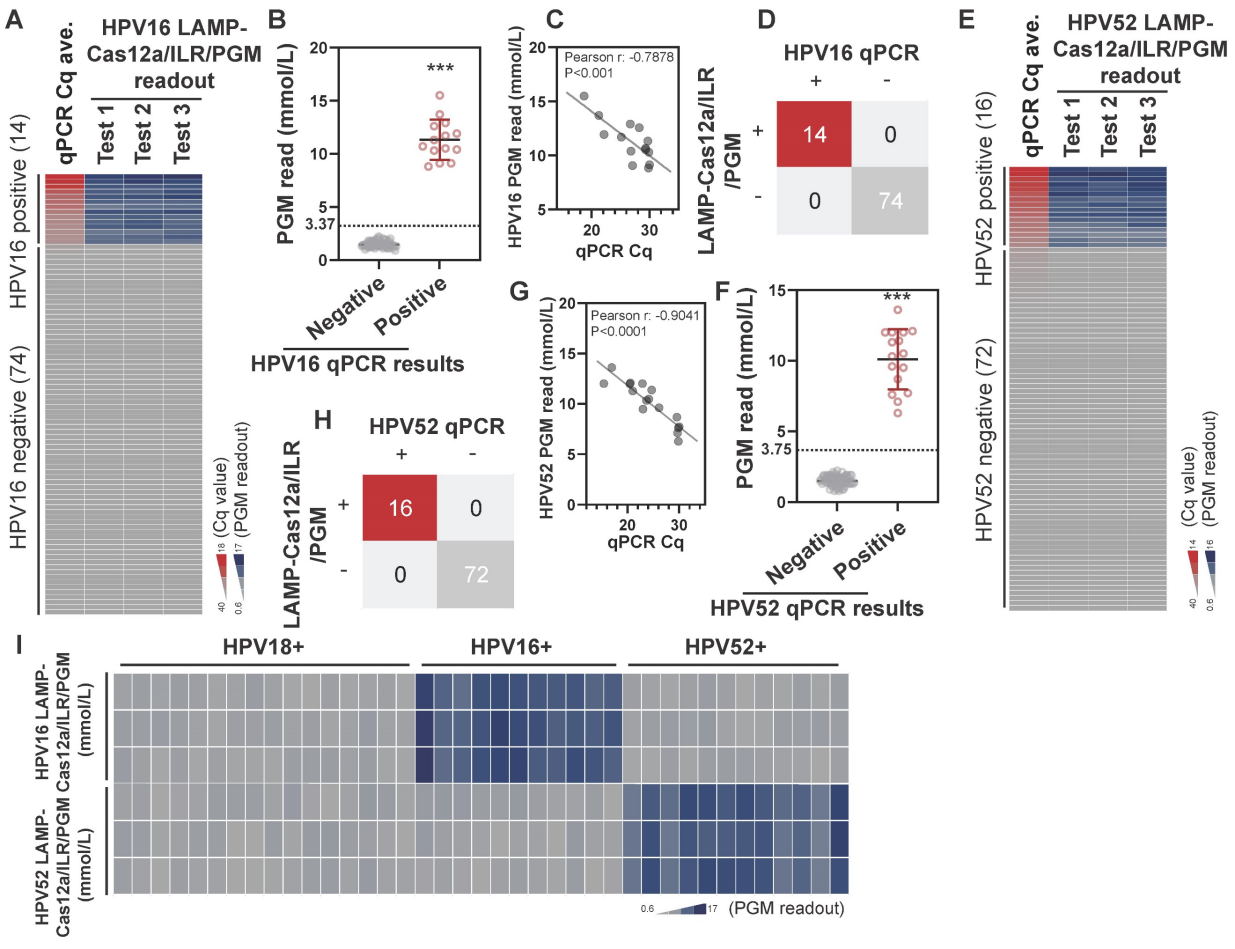
** Detection of HPV16 and HPV52 using the LAMP-Cas12a/ILR/PGM procedure in biological samples.** (A) Heatmap view of qPCR and the HPV16 LAMP-Cas12a/ILR/PGM testing values. Samples are vertically ordered by their qPCR Cq values. (B) PGM readout comparison between the HPV16 qPCR negative and positive groups. Means and SDs are indicated as solid lines. Mean+4SD value of the qPCR negative group was adopted as the threshold for the HPV16 LAMP-Cas12a/ILR/PGM detection. (C) Correlation between the qPCR and PGM readouts in the HPV16 qPCR positive group. (D) Concordance tables between HPV16 qPCR and the LAMP-Cas12a/ILR/PGM detections are shown for clinical samples. (E-H) HPV52 detection was performed in a similar manner as HPV16. (I) Heatmap view of HPV16 and HPV52 LAMP-Cas12a/ILR/PGM detection results across a panel of HPV18, HPV16 and HPV52 positive samples.

Next, we evaluated the potential of this system for detecting other HPV strains. Effective LAMP primers and crRNAs targeting the E6, E7, and E1 genes of HPV16 and HPV52 were identified (see Table S2 and Table S3 for sequence details) through a screening procedure similar to that used for HPV18. These primers and crRNAs were incorporated into the LAMP-Cas12a/ILR/PGM system for the detection of HPV16 and HPV52. In a pool of 88 cytobrush samples with established HPV profiles, increases in LAMP-Cas12a/ILR/PGM readouts were observed for all HPV16-positive samples (n=14) (Figure 6A; see Table S5 for complete results), and the difference in PGM readouts between the positive and negative groups was statistically significant (Figure 6B). In the positive group, a strong correlation was found between the qPCR Cq values and PGM readouts (Figure 6C). The potential of this system for HPV16 diagnosis was evident, with both detection sensitivity and specificity reaching 100% when a similar mean + 4SD threshold was applied (Figure 6D). Notably, comparable detection performance was achieved for HPV52 (Figure 6E-H; see Table S6 for complete results) when the LAMP primers and crRNA were replaced with those targeting HPV52 (Table S2 and Table S3). Furthermore, cross-species specificity was well maintained for both the HPV16 and HPV52 targeted LAMP-Cas12a/ILR/PGM systems (Figure 6I). These findings underscore the adaptability of the LAMP-Cas12a/ILR/PGM system as a stationary testing platform capable of detecting a wide range of targets.

## Conclusions

In this study, we developed a highly efficient nucleic acid detection platform, Cas/ILR/PGM, orchestrated by a solid immobilized enzymatic reporter (ILR) that couples superior nucleic acid detection by Cas12a or Cas13a and efficient signal conversion to glucose abundance via invertase. The entire process is highly operable, consisting of just three tube reactions and one PGM reading. PGM is thus far the most accessible medical devices for widespread use, even in resource-limited settings. Lu's group pioneered the concept of using glucometers for biosensor development 18, 37. Compared to typical portable readouts for Cas12a or Cas13a-based sensing, such as the ANDalyze portable fluorometer, PGM is more portable, cost-effective (with costs under 1% of alternatives), and easy to operate. While its sensitivity may be lower, this limitation can be addressed by the proposed upstream enzymatic signal amplification (Figure 1A).

The integration of the Cas/ILR/PGM system delivered a limit of detection (LOD) comparable to that of high-end laboratory fluorometer-based CRISPR-Cas assessments (Figure 2B-C and Figure S5C-D). This system operates at ambient temperature, eliminating the need for complex devices or professional expertise, thereby making it compatible for cost-effective POCT or at-home diagnosis. However, several limitations exist for the current LAMP-Cas12a/ILR/PGM system for HPV testing, necessitating improvements for its finalization and commercialization. First, this study focused on HPV18, HPV16, and HPV52, specifically targeting their E6, E7, and E1 genes. However, HPV infection can involve a wide range of strains with varying risk levels, and viral integration may occur only in part of the viral genome. Therefore, incorporating multiple primer sets and corresponding crRNAs is essential to enable a more comprehensive assessment of HPV infection status. Second, the prepositioned LAMP-based amplification and Cas12a catalysis require optimal reaction temperatures of 50-65 °C and 37 °C, respectively. Achieving these temperatures with typical portable or household setups, without specialized equipment, presents a challenge. These limitations should be explicitly addressed during the product design phase. We propose the use of a cost-effective isothermal module, such as a battery-powered PTC (positive temperature coefficient) thermal module, to ensure compactness. The hardware, consisting of various reaction compartments and thermal modules, could function as a versatile testing station for a broad range of health-related nucleic acid targets, with on-demand distribution of target-specific reagents. Third, variation in PGM readings was observed across different batches of ILR synthesis (data not shown). We recommend testing and establishing lot- or batch-specific cutoff values for each ILR batch to mitigate batch variability.

The feasibility of the Cas/ILR/PGM system for nucleic acid target testing in biological samples was demonstrated through the detection of HPV18, HPV16, and HPV52 in cultured HPV-positive cell samples and clinical cervical cell specimens. Compared to previously reported CRISPR-Cas-based PGM integrations (Table S1) and other types development in HPV-targeted testing (summarized in Table S7), our LAMP-Cas12a/ILR/PGM system represents a collective advancement in terms of strain diversity, LOD, POCT compatibility, cross-species specificity, and biological sample validation. When cultured cells were used as a model for real-world samples, the LAMP-Cas12a/ILR/PGM system successfully detected ~7 HPV18-positive cells in a mixed cellular sample (Figure 4D-F). More importantly, the system demonstrated excellent performance in detecting HPV18, HPV16, and HPV52 in cervical cell samples, with sensitivities of 95.8% (n=182), 100% (n=88), and 100% (n=88), respectively (Figure 5 and Figure 6). Furthermore, the method exhibited remarkable cross-species specificity (Figure 5F and Figure 6I).

## Materials and Methods

### Construction of the ILR reporters

T_30_ ssDNA and U_30_ ssRNA modified with DBCO TEG on the 5' end (to allow for conjugation to azide-modified invertase) as well as a biotin moiety on the 3' end (to enable attachment to the streptavidin-coated microbeads) were synthesized by Tsingke Biotech.

12 mg of invertase (Sigma-Aldrich Cat. No. I4504) was dissolved in 2 mL of 0.1 M NaHCO_3_ to yield a 100 μM invertase solution. Next, an ~100-fold molar excess of azido-PEG_4_-NHS ester (Aladdin Cat. No. A305020) linkers was introduced to the solution and allowed to react with the lysine residues on the invertase surface for 2 h at room temperature. The azide-functionalized invertase was then passed through a Sephadex desalting gravity column (Sangon, Cat. No. C500090) to remove excess linker. The azide-functionalized invertase in solution was then concentrated by passing through 30-kD spin filters (centrifuged at 7,000 rcf for 5 min).

Azide-functionalized invertase was functionalized with 5'-DBCO TEG-U_30_-biotin-3' RNA or 5'-DBCO TEG-T_30_-biotin-3' DNA oligos, respectively. For this reaction, 200 μL of 10 μM azide-functionalized invertase and 2 equiv. of RNA or DNA were shaken for 15 h in nuclease free solution at room temperature. The invertase-RNA or invertase-DNA conjugates were washed twice (centrifuged at 4,000 rcf for 5 min) and then concentrated (centrifuged at 14,000 rcf for 5 min) by 30 kD spin filters. For fluorescence characterization of the ILR reporter, a small aliquot of invertase-RNA and invertase-DNA conjugates were washed with 20 mM sodium phosphate, 150 mM NaCl, pH 7.2 twice, cross-linked with fluorescein-maleimide, followed by cysteine quenching, and then concentrated.

Microbeads surface was functionalized with invertase-RNA or invertase-DNA conjugates. First, 20 μL of pre-washed streptavidin-coated beads (5 µm, BEAVER Biomed, Cat. No. 22306) were added to 500 μL of nuclease-free PBS containing 0.1% Tween 20. Next, 1 μL of 20 μM invertase-RNA-biotin or invertase-DNA-biotin was introduced, and the solution was gently rotated for 30 min. To separate the unreacted invertase-RNA-biotin or invertase-DNA-biotin, the solution was centrifuged for 1 min at 20,000 rcf. The beads were subsequently washed six times with 1X PBS containing 0.1% Tween 20 and pelleted at the bottom of the tube by centrifugation for 1 min at 20,000 rcf. Finally, the ILR reporter was resuspended in 10 μL of nuclease-free PBS containing 0.1% Tween 20, stored in 4 °C, and used up within two weeks.

The stoichiometry between enzyme, linker, and bead is essential for optimal performance. Re-optimization may be required if altering protocol has to be made. Meanwhile, maintaining the stability and functionality of ILR reporter until the consumption at users' end is essential for the assay. ILR aliquots produced following this protocol can be frozen once at -30 °C for over 9 weeks without obvious integrity and functional decay, according to our data (Figure S4). This single freeze-thaw cycle protocol provides an adequate shelf life from production to consumption.

### Plasmids, cell culture, cytobrush specimen, and sample preparation

The consecutive E6, E7, and E1 (partial) genes' sequences of HPV18, HPV16, and HPV52 were synthesized (Sangon Biotech) and cloned into pCDNA3.1 (-) between EcoRI and NotI sites to form the plasmid pCDNA3.1-HPVE6E7E1 plasmid.

HeLa, HEK-293, and MIA PaCa-2 cells, obtained from Procell Life Science and Technology, were cultured in DMEM supplemented with 10% FBS and 1% penicillin-streptomycin cocktail in a 5% CO_2_ inflated Thermo Forma incubator. Cell density was estimated by hemacytometer-assisted counting. For LOD estimation of the system for cellular samples, serial dilutions (1:10) of HeLa cell suspension (1 million cells/mL) in HEK-293 cell suspension (1 million cells/mL) were generated. 100 μL of cellular mixture was pelleted, followed by a room temperature lysis procedure (Vazyme Biotech, P073) (5 min, in a final 30 μL volume). 2 uL of the lysate was transferred to the subsequent LAMP-Cas12a/ILR/PGM reactions. This procedure resulted in the cellular input series as indicated in Figure 4D-F.

Cytobrush collections in freezing medium were obtained from the Department of Clinical Laboratory at the First Affiliated Hospital of Zhejiang University School of Medicine. 100,000-cell pellets were prepared by hemacytometer-assisted counting and then followed by the same lysis procedure.

The resultant lysate samples can preserve nucleic acid integrity fairly well in a -20 °C refrigerator over the course of a month. These sample preps followed the procedures outlined in the "Loop-Mediated Isothermal Amplification (LAMP)" and "Optimized Cas12a/ILR/PGM Detection Procedure" sections for the full LAMP-Cas12a/ILR/PGM procedure.

### Typical Cas12a and Cas13a collateral cleavage assay (fluorometer based)

Cas13a collateral detection was done as previously reported 38. Cas12a collateral detection was performed on a 384-well plate with a Biotek Synergy Neo2 fluorescent reader. The standard reaction system consisted of 50 nM crRNA (see Table S2 for sequence), 60 nM LbCas12a, 500 nM T_12_ reporter, and w. or w.o. 2 μL of ssDNA target of various concentrations (indicated in specific experiments) in Cas12a nuclease assay buffer (40 mM Tris-HCl, 5 mM MgCl_2_, 100 mM KCl, 1 mM DTT, 5% Glycerol, pH 7.5) in a total volume of 10 μL. All the components were mixed on ice and transferred to a 37 °C pre-warmed plate reader for time-course fluorescence measurements. Specifically, a 485 nm excitation wavelength and a 520 nm emission wavelength were used for FAM fluorescence.

### Loop mediated isothermal amplification (LAMP)

One previous reported and six more LAMP primer sets designed by using the NEB LAMP primer toolset and HPV18 E6, E7, and E1 genes as templates were synthesized at Sangon Biotech (see sequence in Table S3). The reaction mixture was comprised of 1 μL Bst II polymerase (Vazyme Biotech, P702), 6 mM MgSO4, 1.4 mM each dNTP, 1.6 μM each FIP/BIP primers, 0.2 μM each F3/B3 primers, 0.8 μM LF/LB primers, 1X LAMP fluorescence dye (Vazyme Biotech, RP001), 1X isothermal amp buffer, and 2 μL DNA template (either plasmids or cell lysates) in a total volume of 15 μL. The reaction was carried out at 60 °C over the course of 1 h in a Biorad real-time thermal cycler. 2 μL of LAMP product was used as the target for the Cas12a/ILR/PGM system. Similar screening procedure was employed to determine the optimal LAMP primer sets for HPV16 and HPV52 amplification (see sequence in Table S3). It is worth noting that 1 h of LAMP reaction time was used for method development because it adequately allows for completion of all amplifications, target specific or non-specific. Practically, a length of 40 min can be used for time efficiency, as that is adequate for target specific amplifications.

### Optimized Cas12a/ILR/PGM detection procedure

Cas12a collateral reactions, of the same composition as the typical cleavage assay except for 2.5 μL ILR reporter replacing the fluorescence reporter, were performed in PCR tubes at 37 °C with a BIO-RAD PCR instrument for 30 min. The reaction tubes were then set in a magnetic tube stand on ice for 1 min, such that the beads were pelleted at the bottom of the tube and the supernatant (5 μL) could be transferred into a second tube containing 5 μL of 20% sucrose solutions (0.3 M, pH 5.0, acetate buffered). The mixture was subsequently put into a BIO-RAD PCR instrument again for another 20 min at 37 °C. 2 μL of the final product were used for glucose measurement by using a Roche Accu-Chek PGM.

### Cas12a and Cas13a expression and purification

Cas13a expression and purification were conducted as previously reported 38. The pMBP-LbCas12a (addgene#: 113431) expression plasmid was transformed into E. coli BL21 (DE3) competent cells. Starter culture (5 mL) was grown overnight in Terrific Broth and used to inoculate 1 L of TB for growth at 37°C and 200 rpm until OD600 reached 0.5~0.6. Protein expression was subsequently induced with 500 μM IPTG at 18°C for 16 h. Cells were harvested at 5200 g for 15 min at 4°C and stored at -80°C for further purification.

The cell pellet was thawed on ice and resuspended in Lysis buffer (20 mM Tris-HCl, 500 mM NaCl, pH 7.9, 1 mM DTT, 0.5 mM PMSF and 1 mg/mL lysozyme). After sonication and centrifugation, clean supernatant was filtered through a filter of 0.22 μm pore size and purified using Ni-NTA resin. Eluted fractions were incubated with TEV protease overnight at 4°C for MBP tag cleavage. The cleavage mixture was dialyzed and further purified by using Ni-NTA resin for a second time to remove His tag containing proteins.

The flow through was collected, concentrated, and buffer exchanged into storage buffer, followed by concentration determination. Protein was subsequently aliquoted, flash frozen with liquid nitrogen, and transferred to a -80 °C freezer for prolonged storage.

### PGM test strips

Among the many tested PGM devices on the market, only Roche Accu-Chek Active was compatible with glucose detection in clear solution and thus used throughout the work.

### HPV18 detection by the tandem RPA-Cas12a/fluorometer (DETECTR) approach

Three pairs of primers, targeting the same HPV18 loci as the LAMP primer set P123L1, were initially tested for an optimally performing set: HPV18RPA_Fwd gcccgacgagccgaaccacaacgtc and HPV18RPA_Rev cacaccacggacacacaaaggacagg. The reported DETECR procedure was employed with slight modifications 39. Firstly, the mixture of HeLA and HEK-293 cells was processed the same as the LAMP-Cas12a/ILR/PGM method for comparable input of starting materials; Secondly, RPA reaction of 50 µL volume was carried out in a buffer of 50 mM Tris (pH8.0), 100 mM potassium acetate, 2 mM DTT, 5% PEG35K, 14 mM MgCl_2_, 3mM ATP, and 50 mM phosphocreatine. Bsu, UvsX, UVsY, Gp32, and creatine kinase were individually supplemented at final concentrations of 5 U/reaction, 60 ng/µL, 120 ng/µL, 900 ng/µL, and 100 ng/µL. FAM fluorescence at 60 min of the Cas12a cleavage assay was used to determine the experimental LOD.

### LOD calculation and statistical analysis

We employed an empirical approach to determine the limit of detection (LOD) by comparing the analytical responses of blank and low-concentration samples. This approach allowed us to precisely determine the minimum analyte concentration necessary to distinguish its presence from absence. The LOD was defined as the lowest tested analyte concentration that could be reliably detected. GraphPad Prism software was used to perform statistical analysis. At least three independent experiments were performed. Statistical differences between blanks and measurements were determined by an unpaired Student t test. In some experiments, significance is denoted as *, P < 0.05; **, P < 0.01; ***, P < 0.001.

## Supplementary Material

Supplementary methods, figures and tables.
