# Supplementary material for: Coupling CRISPR-Cas and a personal glucose meter with an enzymatic reporter for portable detection of human papillomavirus in biological samples
Source: Theranostics. 2025 Feb 3;15(7):2870–82. doi: 10.7150/thno.106490 (PMC11898299; doi:10.7150/thno.106490)
Supplement: Supplementary file 1 — Supplementary methods, figures and tables. [file thnov15p2870s1.pdf]

Supporting information for

Coupling CRISPR-Cas and a personal glucose meter with an enzymatic reporter for portable detection of human papillomavirus in biological samples

Xuena Zhu[1], Shanshan Wang[2], Yuanyuan Xue[1], Xiaoyan Wang[2], Shaoqi Hu[2], Tingbo Liang[2, 3, 4, 5, 6, *], Wenjun Liu[2, 3, 4,5, 6, *]

[1] Department of Pathology, The First Affiliated Hospital of Zhejiang University School of Medicine, Hangzhou, 310003, China

[2] Zhejiang Provincial Key Laboratory of Pancreatic Disease, The First Affiliated Hospital of Zhejiang University School of Medicine, Hangzhou, 310003, China

[3] Department of Hepatobiliary and Pancreatic Surgery, The First Affiliated Hospital of Zhejiang University School of Medicine, Hangzhou, 310003, China

[4] MOE Joint International Research Laboratory of Pancreatic Diseases, The First Affiliated Hospital of Zhejiang University School of Medicine, Hangzhou, 310003, China

[5] The Innovation Center for the Study of Pancreatic Diseases of Zhejiang Province, Zhejiang University Cancer Center, Hangzhou, 310003, China

[6] Cancer Center, Zhejiang University, Hangzhou, 310058, China.

* Corresponding authors:

Wenjun Liu, Email: WLiu18@zju.edu.cn

Tingbo Liang, Email: LiangTingbo@zju.edu.cn

Contents:

Section A: Supplementary Figures 1-10

Section B. Supplementary Tables 1-7

Section A. Supplementary Figures

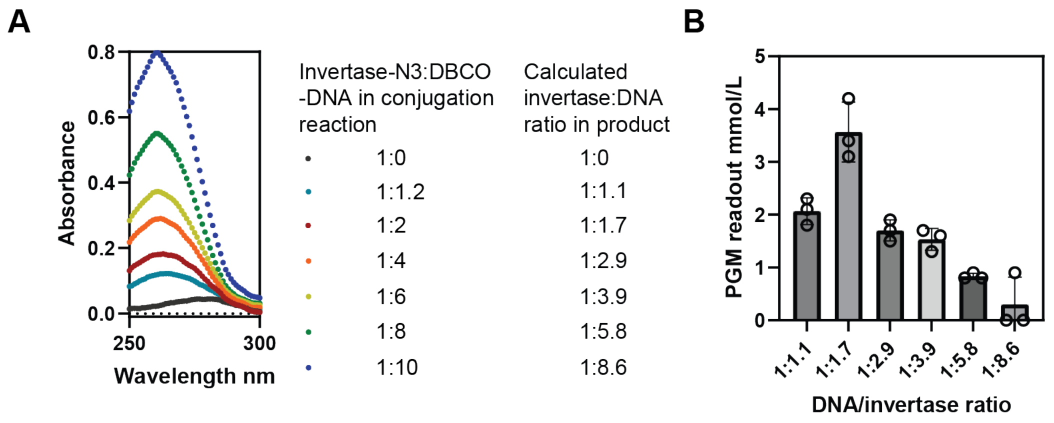

Figure S1. Synthesis of invertase-ssDNA conjugate and optimization of its composition for optimal performance. (A) UV-Vis absorption spectrum illustrating the composition of the invertase-ssDNA conjugate formed during the conjugation reaction, with varying ratios of DBCO-DNA to invertase-N3. (B) PGM readouts of the Cas12a/ILR/PGM system constructed with DNA/invertase conjugates at different ratios, stimulated by 10 pM ssDNA target. A ratio of 1:1.7 was selected for subsequent investigations.

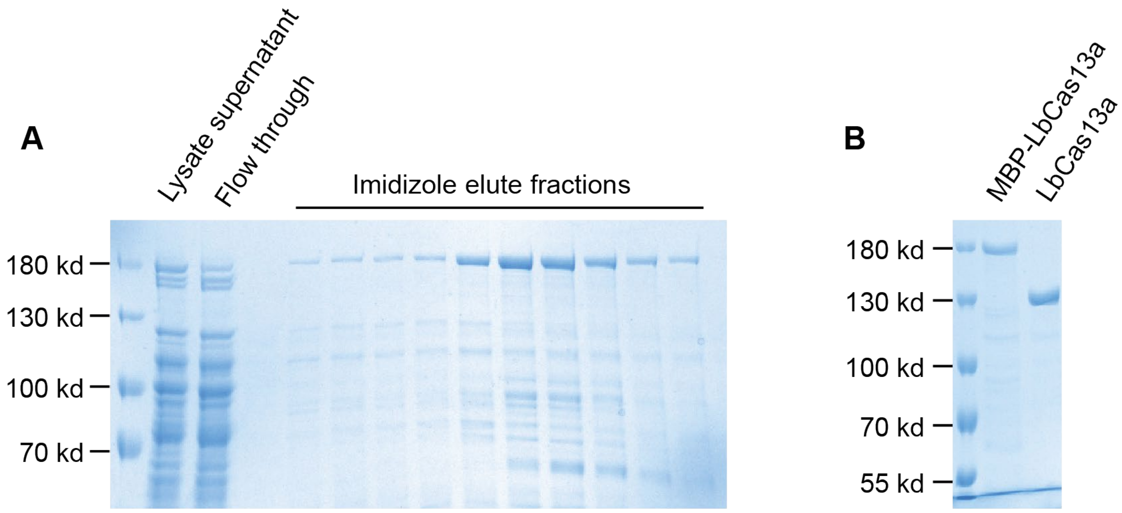

Figure S2. Representative Coomassie blue-stained gels for LbCas12a purification. (A) Filtered lysate supernatant was loaded onto Ni-NTA resins and subsequently washed and eluted with buffers containing 10 mM and 50 mM imidazole, respectively. (B) Elute fractions with high protein content were subjected to TEV cleavage and further purified using Ni-NTA resins, resulting in a highly pure LbCas12a preparation.

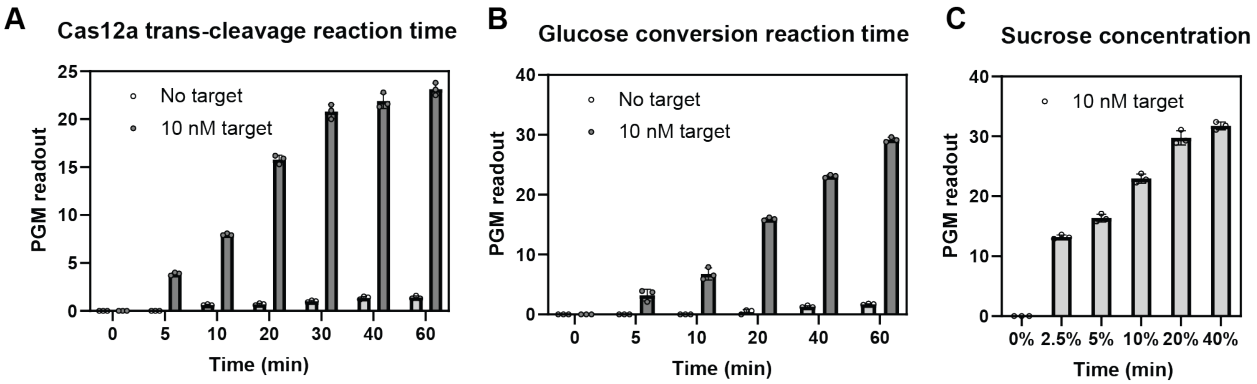

Figure S3. Optimization of detection parameters. (A) A reaction time of 30 min was selected for the Cas12a trans-cleavage reaction, as the signal approached a plateau at this time. (B) A reaction time of 20 min was chosen for the glucose conversion reaction, as it produced negligible noise while yielding a satisfactory PGM readout. (C) A sucrose concentration of 20% was selected for optimal performance.

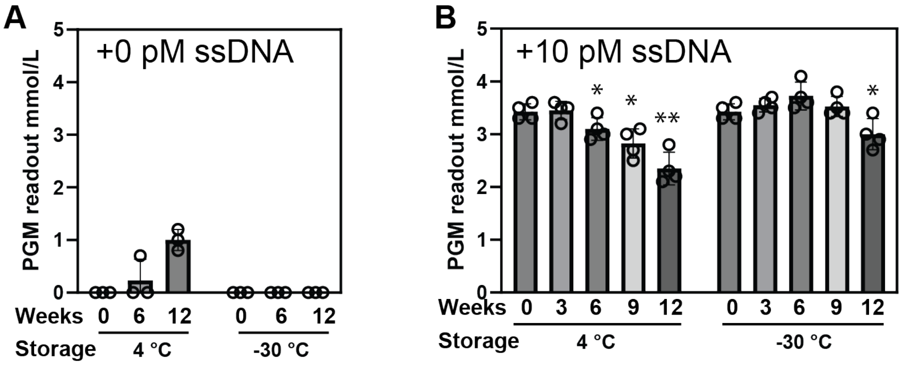

Figure S4. Stability of Cas12a ILR reporter over prolonged storage. Aliquots of ILR, stored at 4 °C or frozen at -30 °C for extended periods, were subjected to the Cas12a/ILR/PGM assay when directly stimulated with 0 pM (A) or 10 pM (B) ssDNA target.

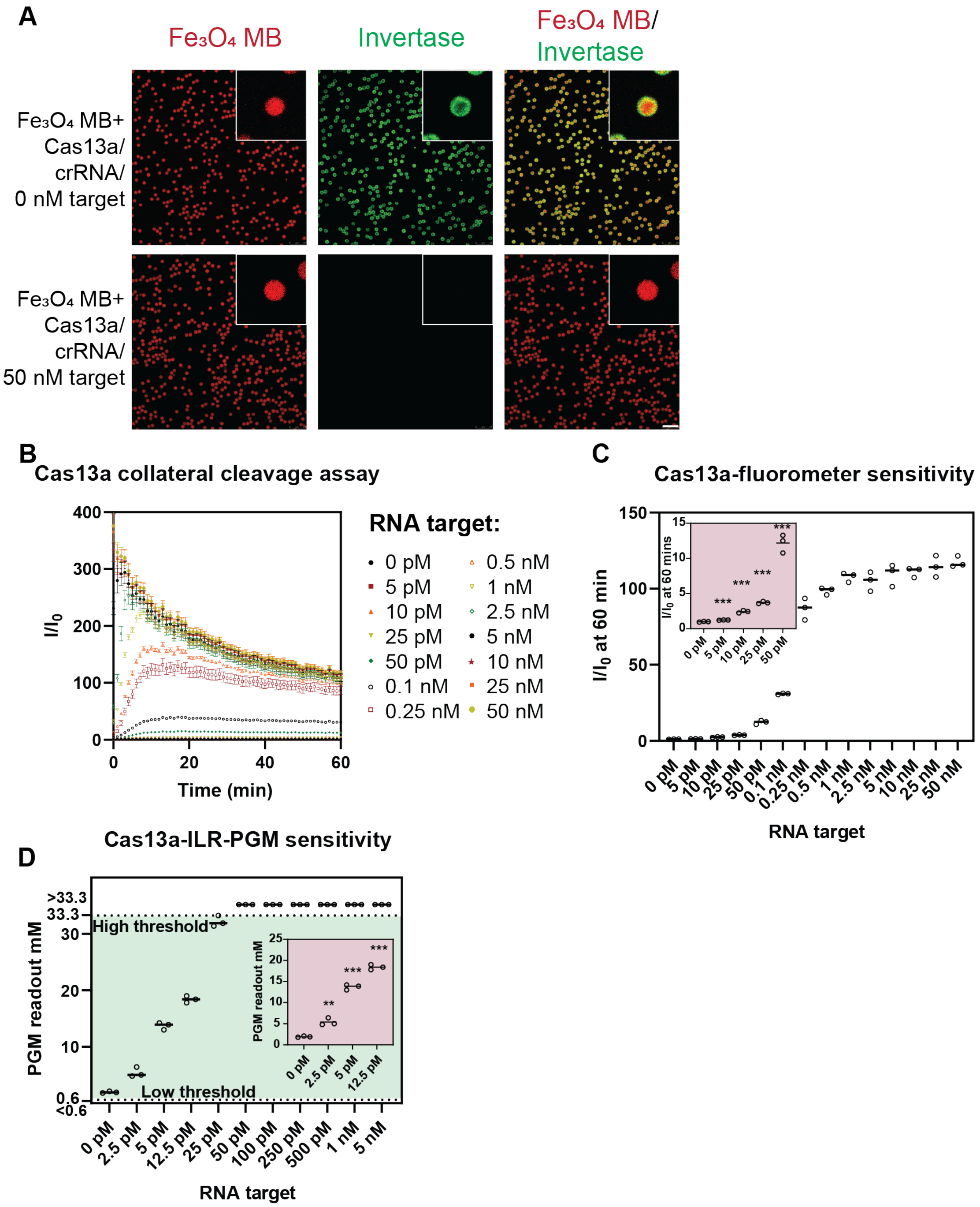

Figure S5. The Cas13a-ILR-PGM system demonstrates satisfactory sensitivity for RNA detection. (A) Confocal fluorescence microscopy to confirm target-dependent release of invertase from the solid phase. The scale bar denotes 25 μm. (B) A typical Cas13a collateral cleavage assay, recorded using a BioTek Synergy Neo2 fluorometer. (C) LOD determination indicates an empirical LOD of 5 pM. (D) PGM readouts in response to serial dilution of RNA target using the Cas13a/ILR/PGM system, showing an improved LOD of 2.5 pM.

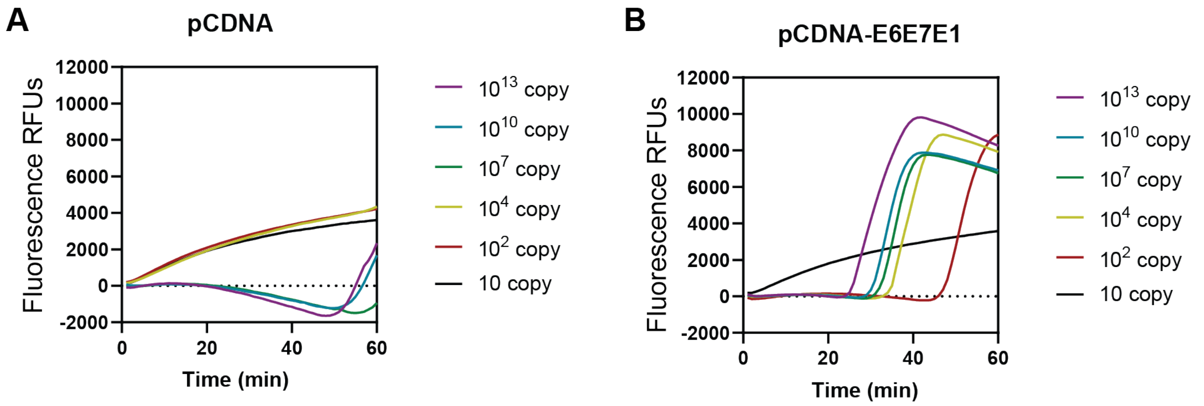

Figure S6. Real-time fluorescence of LAMP reactions with pCDNA3.1 (A) and pCDNA-E6E7E1 (B) plasmid templates.

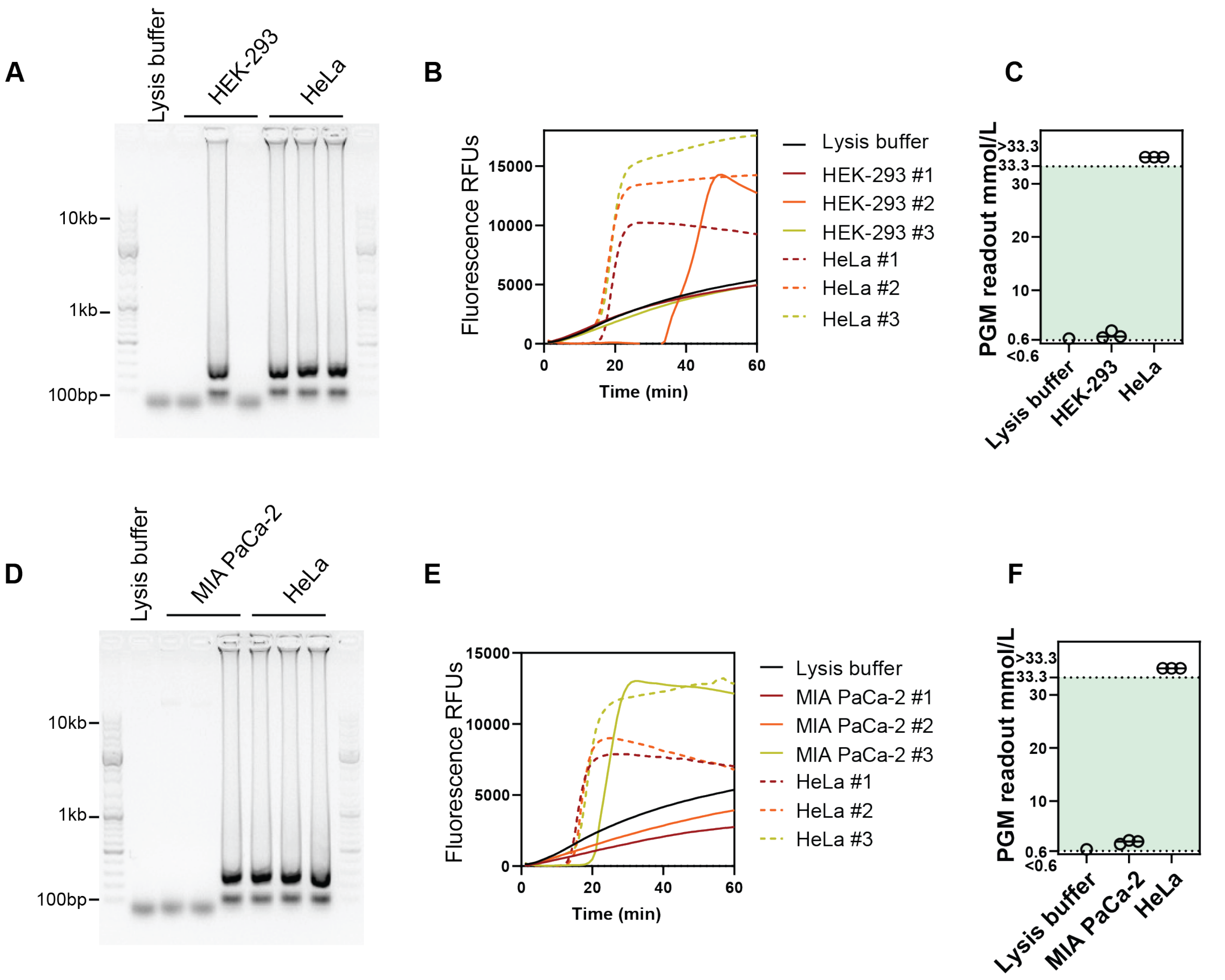

Figure S7. HeLa cells tested positive using the LAMP-Cas12a/ILR/PGM system. (A and D) Agarose gel showing the products of LAMP reactions with HEK-293, HeLa, and MIA PaCa-2 cell lysates. (B and E) Real-time fluorescence measurement of the LAMP reactions. (C and F) Subsequent Cas12a/ILR/PGM readouts of the LAMP products from (A-B) and (D-E), respectively.

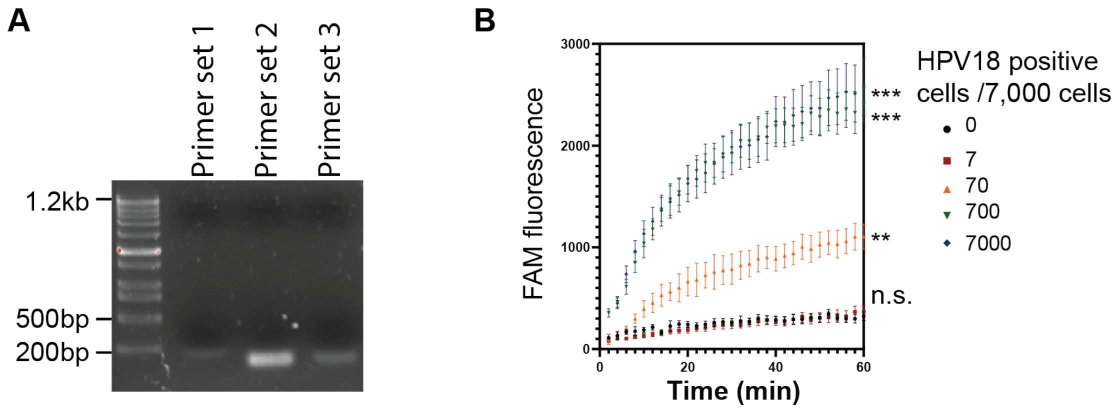

Figure S8. RPA-Cas12a/fluorometer-based detection of HPV18 gene in cultured cell samples. (A) Different primer sets were tested for optimal RPA amplification of the HPV18 gene. (B) Real-time monitoring of emerging FAM fluorescence in the tandem RPA-Cas12a/fluorometer assay (DITECTR).

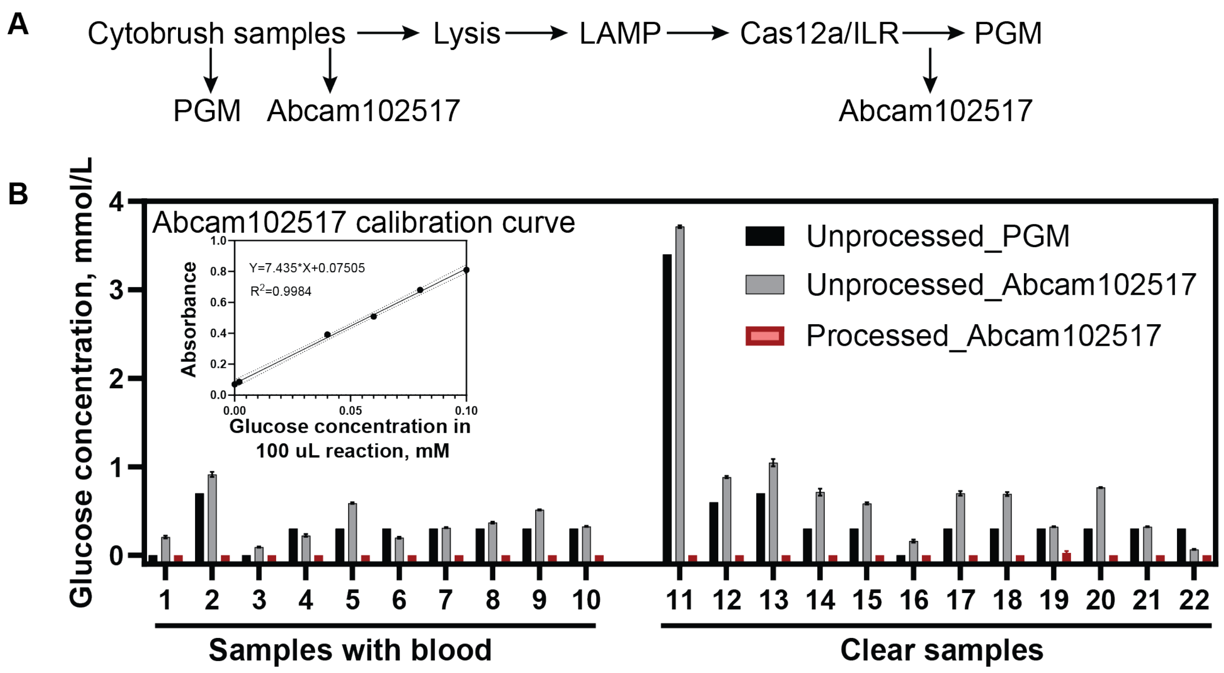

Figure S9. Intrinsic glucose in cytobrush samples shows negligible interference with the LAMP-Cas12a/ILR/PGM system. (A) Unprocessed cytobrush samples and intermediate reaction mixtures were subjected to glucose measurement. In addition to PGM, an Abcam glucose assay kit was used to quantify glucose at concentrations ranging from 0.02 to 10 mM. (B) Glucose concentrations in all 22 samples, with or without visible blood, decreased to negligible levels after processing. Note: all PGM reads of “E-2” and “Lo” were assigned values of 0 and 0.3 mmol/L, respectively. For the Abcam assay, all absorbance values below the Y-intercept of the calibration curve were assigned a glucose concentration of 0 mmol/L.

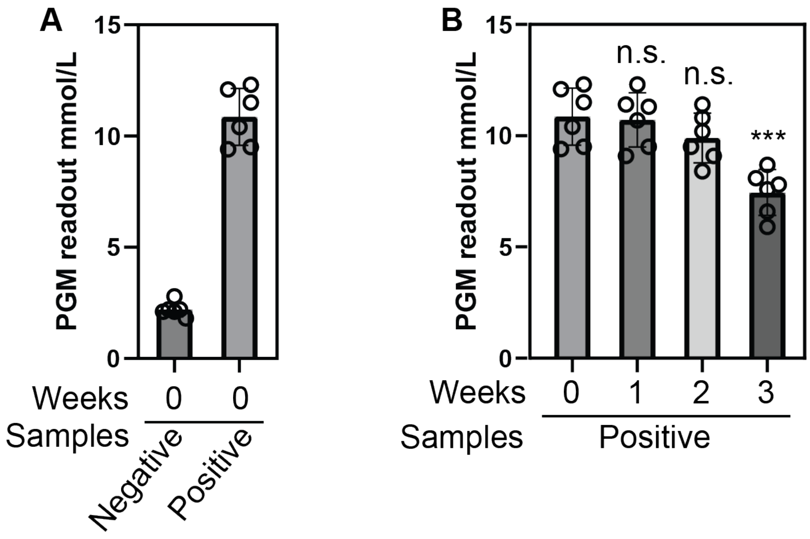

Figure S10. Stability and reproducibility of the HPV18 LAMP-Cas12a/ILR/PGM system in its ready-to-use format. (A) Individual tubes of LAMP reaction mixture, Cas12a/ILR, and sucrose solutions at their complete compositions were prepared and stored at -30 °C for the indicated duration. The reagents were then used to test pellets from a pair of randomly selected negative and positive samples, evaluating system reproducibility and stability. The testing revealed a relative standard deviation (RSD) of 14.9% for the negative sample and 11.8% for the positive sample, indicating good reproducibility. (B) Significant signal degradation was observed in reagents stored for longer than 2 weeks.

Section B. Supplementary Tables

Table S1. CRISPR-Cas and PGM integration for nucleic acid detection

Taget	LOD	Verification in biological samples	Sensitivity and specificity	Reference PMID
HPV18, HPV16, and HPV52	~7 HPV18 positive cells per reaction	Cervical cellular samples (n=182, 88 and 88)	95.8% and 100%; 100% and 100%; 100% and 100%	This work
CRISPR-Cas PGM integration for nucleic acid detection	57 pM	Nucleic acid molecules	NA	39171545
HIV (DNA and RNA)	43 copies and 200 copies per test, respectively.	Plasma samples (n=15)	85.7% and 100%	36762838
HIV-related DNA or SARS-CoV-2 N gene	11.0 fM and 50 copies/μL, respectively.	Nucleic acid molecules	NA	35689948
miRNA-21 and miRNA-205	2.4 and 1.1 pM, respectively.	Nucleic acid molecules	NA	34279073
Alpha-fetoprotein	10 ng/mL	Clinical serum samples (n=11)	100% and 100%	37303825
N-gene and PCB77	6 fM and 3.2×10-5 μg/L, respectively.	Nucleic acid molecules	NA	36220300
SARS-CoV-2 N-gene	1.8×10^4 virus particles (VPs) per μL	Throat swab samples (n=6)	100% and 100%	34276930
SARS-CoV-2	10 copies/μl	Throat swab samples (n=11)	NA	33410130
N gene	0.15 pM	Nucleic acid molecules	NA	38503121

Table S2. Nucleic acid sequence

Name	Sequence	Figures
P81L4 crRNA (HPV18)		
	UAAUUUCUACUAAGUGUAGAUccagcccgacgagccgaacc (Cas12a)	3E
P60L1 crRNA (HPV18)		
	UAAUUUCUACUAAGUGUAGAUaucguuuucuuccucugagu (Cas12a)	3E
P38L10 crRNA (HPV18)		
	UAAUUUCUACUAAGUGUAGAUauuuuggggcucuaaaugca (Cas12a)	3E
P123L1 crRNA (HPV18)		
	UAAUUUCUACUAAGUGUAGAUuacuacuagcucaauucuggc (Cas12a)	3E, 4B, 4C 4F, 5, 6, S7C and F, S10
HPV16 crRNA		
	UAAUUUCUACUAAGUGUAGAUtacgcacaaccgaagcgtag (Cas12a)	6
HPV52 crRNA		
	UAAUUUCUACUAAGUGUAGAUagguugcagaucuaauauau (Cas12a)	6
miR21 crRNA		
	UAAUUUCUACUAAGUGUAGAUucaacaucagucugauaagcua (Cas12a)	1D-F, 2, S2
	GAUUUAGACUACCCCAAAAACGAAGGGGACUAAAACucaacaucagucugauaagcua (Cas13a)	S3
miR21 target		
	TAGCTTATCAGACTGATGTTGA (DNA, Cas12a)	1D-F, 2, S3
	UAGCUUAUCAGACUGAUGUUGA (RNA, Cas13a)	S5
T12 ssDNA reporter		
	FAM-TTTTTTTTTTTT-BHQ1	2A-B, S8B
U5 ssRNA reporter		
	FAM-UUUUU-BHQ1	S5
pCDNA3.1-E6E7E1		
	HPV18 E6, E7 and E1 genome as revealed on pave.niaid.nih.gov was synthesized and cloned into pCDNA3.1	3D-E, 4A-C, S6

Note: denoted red is the direct repeat regions

Table S3. Selection of LAMP primer sequence

Target	Primer name	Primer sequence
HPV18	PDatV	
	PDatV_F3	ACAAGCTACCTGATCTGTG
	PDatV_B3	ATCGTCGTTTTTCATTAAGGT
	PDatV_FIP	CGGTATACTGTCTCTATACACCACATTTTCACGGAACTGAACACTTCA
	PDatV_BIP	TATTCAGACTCTGTGTATGGAGACATTTTAAGTTTTTCTGCTGGATTCAAC
	PDatV_LF	CTGTAAGTTCCAATACTGTCTTGCA
	PDatV_LB	TTTATTAATAAGGTGCCTGCGGTGC
	P81L4	
	P81L4_F3	TGAAATTCCGGTTGACCT
	P81L4_B3	CTGGAATGCTCGAAGGTC
	P81L4_FIP	CGGGCTGGTAAATGTTGATGATTAACACGAGCAATTAAGCGAC
	P81L4_BIP	CCGAACCACAACGTCACACAACTAGCTCAATTCTGGCTT
	P81L4_LF	TCATCGTTTTCTTCCTCTGA
	P81L4_LB	GTGTATGTGTTGTAAGTGTG
	P82	
	P82_F3	TGAAATTCCGGTTGACCT
	P82_B3	CTGGAATGCTCGAAGGTC
	P82_FIP	CGGGCTGGTAAATGTTGATGATTAACACGAGCAATTAAGCGAC
	P82_BIP	CCGAACCACAACGTCACACAACTAGCTCAATTCTGGCTT
	P60L1	
	P60L1_F3	CATTGCAAGACATTGTATTGC
	P60L1_B3	GCTGAGCTTTCTACTACTAGC
	P60L1_FIP	TCCTCTGAGTCGCTTAATTGCTGAGCCCCAAAATGAAATTCC
	P60L1_BIP	ATCAACATTTACCAGCCCGACGTCAATTCTGGCTTCACACT
	P60L1_LB	AGCCGAACCACAACGTCAC
	P73L1	
	P73L1_F3	CCAAAATGAAATTCCGGTTGA
	P73L1_B3	GTCGTCTGCTGAGCTTTC
	P73L1_FIP	CGGGCTGGTAAATGTTGATGATTAACCTTCTATGTCACGAGCAATT
	P73L1_BIP	CCGAACCACAACGTCACACATAGCTCAATTCTGGCTTCA
	P73L1_LF	CGTTTTCTTCCTCTGAGTCGCTT
	P38L10	
	P38L10_F3	AACGACGCAGAGAAACAC
	P38L10_B3	TGTGTGACGTTGTGGTTC
	P38L10_FIP	TGGGGCTCTAAATGCAATACAATGGTATAATATTAAGTATGCATGGACC
	P38L10_BIP	GTTGACCTTCTATGTCACGAGCAATGGTAAATGTTGATGATTAACTCC
	P38L10_LB	CGACTCAGAGGAAGAAAACGATGA
	P123L1	
	P123L1_F3	CGAACCACAACGTCACAC
	P123L1_B3	CCTTCTGGATCAGCCATTG
	P123L1_FIP	TCGTCTGCTGAGCTTTCTACTACGTTGTGTATGTGTTGTAAGTGTG
	P123L1_BIP	CTTCGAGCATTCCAGCAGCTTTGCTTACTGCTGGGATG
	P123L1_LB	GTTTCTGAACACCCTGTCCTTTGT
HPV16	HPV16_LAMP	
	HPV16_LAMP_F3	AGCCCATTACAATATTGTAACC
	HPV16_LAMP_B3	CATCCCGTACCCTCTTCC
	HPV16_LAMP_FIP	CGAATGTCTACGTGTGTGCTTTTTTTGTTGCAAGTGTGACT
	HPV16_LAMP_BIP	TGGGCACACTAGGAATTGTGTTTGGTACCTGCAGGATCA
	HPV16_LAMP_LB	CCCCATCTGTTCTCAGAAACCATAA
HPV52	HPV52_LAMP	
	HPV52_LAMP_F3	GTTCAGAGTGTTGGAGACC
	HPV52_LAMP_B3	ACAATGTAGTAATTGCTTGTGG
	HPV52_LAMP_FIP	GTCAGTTGTTTCAGGTTGCAGATCCTGTGACCCAAGTGTAACG
	HPV52_LAMP_BIP	TATGAGCAATTAGGTGACAGCTCACTTGTTCTGCTTGTCCATC
	HPV52_LAMP_LF	TGCTTTGTCTCCACGCATGA

Note: Primer set 7 was advantageous in low self-amplification and used in later work

Table S4. Clinical sample testing results by HPV18 qPCR and LAMP-Cas12a/ILR/PGM

Patient #	Clinical HPV18 status	Self conducted HPV18 qPCR (Cq value)				HPV18 LAMP-Cas12a/ILR/PGM					
		Test 1	Test 2	Test 3	Average	PGM reads (n=3)			Average	STDEV	LAMP-Cas12a/ILR/PGM positive or not
1	N	36.31	38.69		37.50	1.4	1.3	1.4	1.4	0.06	N
2	N	34.80	38.25		36.53	1.8	1.3	1.4	1.5	0.26	N
3	N	33.45	36.43		34.94	1.7	1.3	1.3	1.4	0.23	N
4	N	34.18	36.46		35.32	1.6	1	1.3	1.3	0.30	N
5	N	33.29	36.27		34.78	1.4	1.2	1.2	1.3	0.12	N
6	N	33.54	36.38		34.96	1.3	1.4	1.3	1.3	0.06	N
7	N	32.95	38.84		35.90	1.1	0.9	1.1	1.0	0.12	N
8	P	20.57	21.21	22.58	21.45	12.7	12.6	13.2	12.8	0.32	P
9	N	34.42	N/A		34.42	1.1	0.9	1.3	1.1	0.20	N
10	N	33.88	36.66		35.27	1.4	1.2	0.8	1.1	0.31	N
11	N	32.83	38.71	N/A	35.77	1.6	1.4	1.4	1.5	0.12	N
12	N	35.80	N/A		35.80	1.4	1.3	1.3	1.3	0.06	N
13	N	38.39	N/A		38.39	1	0.9	1.3	1.1	0.21	N
14	N	36.09	36.96		36.52	1.3	1.2	1.1	1.2	0.10	N
15	N	33.84	36.57		35.21	1.1	0.8	1.2	1.0	0.21	N
16	N	34.75	39.40		37.07	1.4	0.9	1.2	1.2	0.25	N
17	N	34.24	N/A		34.24	1.6	1.3	1.4	1.4	0.15	N
18	N	33.76	36.55		35.16	1.3	0.9	0.9	1.0	0.23	N
19	N	33.04	37.13		35.09	1.1	1.3	1.1	1.2	0.12	N
20	N	33.85	38.62		36.23	1.4	1	1.3	1.2	0.21	N
21	N	32.85	36.79		34.82	1.1	1.2	0.8	1.0	0.21	N
22	N	32.15	38.79		35.47	1.6	1.1	0.9	1.2	0.36	N
23	N	35.25	N/A		35.25	1.3	1.2	1.3	1.3	0.06	N
24	N	33.78	N/A		33.78	1	0.9	1.2	1.0	0.15	N
25	N	33.57	36.87		35.22	1.2	1.2	0.9	1.1	0.17	N
26	N	33.51	38.26		35.88	1.4	1.2	1.1	1.2	0.15	N
27	N	33.69	N/A		33.69	1	1	1.1	1.0	0.06	N
28	N	33.98	37.35		35.67	1.1	0.9	1	1.0	0.10	N
29	N	32.55	36.22		34.38	0.8	1.2	1.3	1.1	0.26	N
30	N	33.13	36.14		34.64	0.9	0.8	1.1	0.9	0.15	N
31	N	33.01	36.43		34.72	1.2	1	0.9	1.0	0.15	N
32	N	33.14	38.10		35.62	1.2	1.2	1.3	1.2	0.06	N
33	N	33.21	36.38	35.27	34.95	1.3	1.3	0.9	1.2	0.23	N
34	P	25.71	26.77	27.24	26.57	9.3	8.4	10.4	9.4	1.00	P
35	N	33.43	36.89		35.16	1.1	0.9	0.8	0.9	0.15	N
36	N	33.28	37.49		35.39	1	0.9	1	1.0	0.06	N
37	N	33.86	38.05		35.96	1.1	1	0.9	1.0	0.10	N
38	N	32.63	N/A		32.63	0.9	0.9	1	0.9	0.06	N
39	N	32.59	35.17		33.88	1.3	0.9	1.1	1.1	0.20	N
40	N	32.46	37.07		34.77	0.9	0.7	0.8	0.8	0.10	N
41	N	34.54	N/A		34.54	1.1	1.3	1.1	1.2	0.12	N
42	N	32.91	36.27		34.59	0.8	1.1	0.9	0.9	0.15	N
43	N	35.58	N/A		35.58	1.1	0.9	1	1.0	0.10	N
44	N	34.36	36.77		35.56	1.2	0.9	1.0	1.0	0.15	N
45	N	33.75	36.95		35.35	1.1	0.8	0.9	0.9	0.15	N
46	N	34.28	37.83		36.06	0.8	1.0	1.0	0.9	0.12	N
47	N	33.07	37.56		35.31	0.9	0.8	0.7	0.8	0.10	N
48	N	33.44	35.37		34.40	1.3	1.1	1.3	1.2	0.12	N
49	N	32.77	36.82		34.79	0.9	1.1	1.1	1.0	0.12	N
50	N	33.91	37.07		35.49	1.3	1.2	1.2	1.2	0.06	N
51	N	33.87	36.90		35.38	1.6	1.1	1.3	1.3	0.25	N
52	N	33.01	38.96		35.98	1.4	1.1	1.2	1.2	0.15	N
53	N	33.73	38.36		36.04	1.1	1.3	1.4	1.3	0.15	N
54	N	33.45	35.49		34.47	1.4	0.9	0.8	1.0	0.32	N
55	N	33.12	35.48		34.30	1.7	1.1	0.9	1.2	0.42	N
56	N	33.38	39.83		36.61	1.4	1.2	0.9	1.2	0.25	N
57	N	38.55	36.04		37.29	1.3	1.1	1.3	1.2	0.12	N
58	N	33.05	39.36		36.20	1.1	0.9	0.8	0.9	0.15	N
59	N	30.62	30.90	33.90	31.81	1.7	1.3	1.1	1.4	0.31	N
60	N	34.33	39.16	N/A	36.74	1.0	0.9	0.7	0.9	0.15	N
61	N	33.71	36.57	N/A	35.14	1.4	1.3	1.4	1.4	0.06	N
62	N	29.56	30.17	31.41	30.38	1.6	1.2	1.3	1.4	0.21	N
63	N	33.17	38.28		35.72	1.3	1.2	1.2	1.2	0.06	N
64	N	34.57	N/A		34.57	0.9	0.8	1.1	0.9	0.15	N
65	N	33.87	39.66		36.76	1.0	0.9	1.1	1.0	0.10	N
66	N	35.31	N/A		35.31	1.1	1.0	0.9	1.0	0.10	N
67	N	35.03	36.72		35.88	1.2	1.2	1.1	1.2	0.06	N
68	N	34.28	N/A		34.28	1.6	1.1	0.9	1.2	0.36	N
69	N	32.79	38.35		35.57	1.3	1.2	0.9	1.1	0.21	N
70	N	34.70	N/A		34.70	1.1	0.9	0.9	1.0	0.12	N
71	N	35.04	39.74		37.39	1.2	0.9	0.8	1.0	0.21	N
72	N	34.98	37.76		36.37	1.4	1.2	1.0	1.2	0.20	N
73	N	37.28	37.69		37.49	1.3	1.1	1.0	1.1	0.15	N
74	N	34.22	37.38		35.80	1.0	0.9	1.2	1.0	0.15	N
75	N	33.40	37.43		35.42	1.4	1.2	1.1	1.2	0.15	N
76	N	33.32	N/A		33.32	1.1	1.2	1.2	1.2	0.06	N

77	N	34.86	N/A		34.86	1.6	1.1	1.1	1.3	0.29	N
78	N	34.10	N/A		34.10	1.1	0.9	1.1	1.0	0.12	N
79	N	33.62	39.25		36.44	1.1	1.0	0.9	1.0	0.10	N
80	N	34.13	N/A		34.13	1.2	1.1	0.8	1.0	0.21	N
81	N	38.01	N/A		38.01	1.4	1.1	0.9	1.1	0.25	N
82	N	33.10	35.63		34.37	1.2	1.1	1.2	1.2	0.06	N
83	N	34.69	37.75		36.22	1.4	1.3	1.2	1.3	0.10	N
84	N	34.07	39.72		36.89	1.1	1.3	1.2	1.2	0.10	N
85	N	34.04	37.33		35.69	1.4	0.9	1.1	1.1	0.25	N
86	N	35.01	37.68		36.34	1.1	0.8	0.9	0.9	0.15	N
87	N	37.63	N/A		37.63	1.5	1.1	0.7	1.1	0.40	N
88	N	34.84	N/A		34.84	1.8	1.3	0.8	1.3	0.50	N
89	P	29.37	29.59		29.48	1.4	1.3	1.2	1.3	0.10	N
90	N	36.42	N/A	37.39	36.91	1.8	1.6	1.4	1.6	0.20	N
91	P	22.40			22.40	11.5	8.3	11.0	10.3	1.72	P
92	P	25.71			25.71	11.7	8.3	12.0	10.7	2.06	P
93	N	35.75			35.75	1.1	1.2	1.1	1.1	0.06	N
94	N	32.69			32.69	1.3	0.9	1.2	1.1	0.21	N
95	N	N/A	34.01	38.86	36.44	2.1	1.7	1.5	1.8	0.31	N
96	N	32.06	34.63	33.20	33.30	0.6	1.4	1.6	1.2	0.53	N
97	N	35.49	33.08	33.09	33.88	2.4	2.1	1.5	2.0	0.46	N
98	P	23.42	23.35	23.60	23.46	7.8	9.3	6.4	7.8	1.45	P
99	N	36.74	39.72	32.76	36.41	2.8	1.5	0.9	1.7	0.97	N
100	N	31.67	30.70	32.70	31.69	0.8	0.6	1.3	0.9	0.36	N
101	P	24.72	24.61	24.81	24.71	10.4	10.3	11.8	10.8	0.84	P
102	N	33.27	N/A	32.07	32.67	2.0	1.6	1.9	1.8	0.21	N
103	N	35.10	33.81	N/A	34.45	2.1	0.9	1.2	1.4	0.62	N
104	N	31.20	33.30	33.13	32.54	1.5	1.2	0.7	1.1	0.40	N
105	N	36.39	32.48	32.99	33.96	1.7	1.3	2.1	1.7	0.40	N
106	P	24.24	24.46	24.25	24.32	12.4	11.5	12.0	12.0	0.45	P
107	N	33.08	34.94	33.66	33.89	1.9	1.6	1.3	1.6	0.30	N
108	N	34.89	34.01	31.69	33.53	1.8	2.2	1.4	1.8	0.40	N
109	N	33.85	34.39	32.70	33.65	1.3	1.5	1.6	1.5	0.15	N
110	P	18.84	18.73	19.04	18.87	12.5	13.1	13.8	13.1	0.65	P
111	N	32.53	33.15	31.16	32.28	1.5	1.9	1.2	1.5	0.35	N
112	P	27.19	27.24	27.23	27.22	7.5	6.9	8.4	7.6	0.75	P
113	P	20.17	20.48	20.27	20.31	12.5	13.2	11.9	12.5	0.65	P
114	P	28.88	28.81	28.57	28.75	9.2	8.6	8.0	8.6	0.60	P
115	P	24.16	24.01	24.11	24.09	11.6	12.1	11.9	11.9	0.25	P
116	N	39.31	35.21	34.71	36.41	1.3	1.5	1.2	1.3	0.15	N
117	N	33.92	31.43	30.64	32.00	1.1	0.6	1.7	1.1	0.55	N
118	N	33.81	33.63	31.06	32.83	1.2	0.9	1.4	1.2	0.25	N
119	N	31.44	31.29	30.09	30.94	1.8	2.1	1.4	1.8	0.35	N
120	N	31.26	31.39	32.44	31.70	1.3	2.1	1.7	1.7	0.40	N
121	N	31.00	32.46	30.61	31.36	1.7	2.3	1.9	2.0	0.31	N
122	N	30.67	32.07	31.44	31.40	1.9	1.4	1.3	1.5	0.32	N
123	P	17.30	17.31	17.13	17.25	13.5	12.9	13.4	13.3	0.32	P
124	P	29.64	29.15	29.59	29.46	5.9	7.2	6.8	6.6	0.67	P
125	P	29.38	29.23	28.41	29.00	7.4	7.3	6.8	7.2	0.32	P
126	P	27.86	28.02	27.36	27.75	8.1	8.9	7.8	8.3	0.57	P
127	P	22.69	22.45	22.28	22.47	9.9	9.3	9.0	9.4	0.46	P
128	N	32.27	32.08	33.11	32.49	0.8	1.6	1.8	1.4	0.53	N
129	P	18.71	18.49	18.49	18.56	14.2	13.9	12.8	13.6	0.74	P
130	P	24.33	24.23	24.14	24.24	7.9	9.5	8.3	8.6	0.83	P
131	P	17.96	17.49	17.79	17.75	12.9	13.5	12.5	13.0	0.50	P
132	N	N/A	32.31	32.70	32.50	1.7	1.4	1.9	1.7	0.25	N
133	N	N/A	39.32	N/A	39.32	2.9	2.1	1.9	2.3	0.53	N
134	N	N/A	34.84	35.08	34.96	2.3	2.6	1.8	2.2	0.40	N
135	N	35.27	N/A	N/A	35.27	1.6	0.9	0.8	1.1	0.44	N
136	N	N/A	N/A	N/A	N/A	0.9	1.0	1.3	1.1	0.21	N
137	N	N/A	33.79	N/A	33.79	1.9	2.1	2.2	2.1	0.15	N
138	N	N/A	32.56	N/A	32.56	1.8	1.9	1.5	1.7	0.21	N
139	N	N/A	N/A	N/A	N/A	1.0	1.3	1.7	1.3	0.35	N
140	N	N/A	35.70	N/A	35.70	1.8	2.1	0.9	1.6	0.62	N
141	N	35.15	N/A	N/A	35.15	0.8	0.9	1.6	1.1	0.44	N
142	N	N/A	N/A	34.16	34.16	1.2	2.1	1.5	1.6	0.46	N
143	N	33.15	37.10	N/A	35.12	1.8	1.3	0.9	1.3	0.45	N
144	N	N/A	N/A	N/A	N/A	1.1	1.4	1.8	1.4	0.35	N
145	N	N/A	N/A	35.00	35.00	1.7	1.5	1.3	1.5	0.20	N
146	N	N/A	N/A	N/A	N/A	0.8	1.7	1.9	1.5	0.59	N
147	N	34.09	39.52	35.17	36.26	1.6	0.9	1.4	1.3	0.36	N
148	N	N/A	37.15	N/A	37.15	2.1	1.6	1.9	1.9	0.25	N
149	N	N/A	N/A	N/A	N/A	1.9	1.3	1.2	1.5	0.38	N
150	N	31.95	N/A	N/A	31.95	1.3	1.8	2.3	1.8	0.50	N
151	N	N/A	N/A	N/A	N/A	1.4	1.8	1.9	1.7	0.26	N
152	N	N/A	N/A	N/A	N/A	0.8	1.3	1.6	1.2	0.40	N
153	N	N/A	N/A	N/A	N/A	1.2	1.3	2.8	1.8	0.90	N
154	N	N/A	39.41	36.50	37.95	1.8	0.7	1.5	1.3	0.57	N
155	N	N/A	N/A	N/A	N/A	1.9	1.7	2.1	1.9	0.20	N
156	N	35.34	N/A	N/A	35.34	1.9	2.4	2.1	2.1	0.25	N
157	N	N/A	N/A	33.50	33.50	1.8	0.9	1.7	1.5	0.49	N
158	N	N/A	N/A	N/A	N/A	1.0	1.8	1.2	1.3	0.42	N

159	P	14.27	14.26	14.27	13.9	12.1	12.4	12.8	0.96	P
160	P	17.65	17.46	17.55	12.2	12.7	13.7	12.9	0.76	P
161	N	N/A	N/A	N/A	1.4	1.8	2.2	1.8	0.40	N
162	N	N/A	34.56	34.56	1.2	1.9	1.8	1.6	0.38	N
163	N	N/A	N/A	N/A	1.5	2.3	0.9	1.6	0.70	N
164	N	33.47	N/A	33.47	1.2	2.2	1.7	1.7	0.50	N
165	N	34.32	N/A	34.32	1.1	1.5	1.1	1.2	0.23	N
166	N	N/A	N/A	N/A	1.0	1.4	1.1	1.2	0.21	N
167	N	N/A	N/A	N/A	2.0	1.7	1.3	1.7	0.35	N
168	N	N/A	N/A	N/A	1.3	1.9	0.9	1.4	0.50	N
169	N	30.84	30.30	30.57	1.8	1.7	2.4	2.0	0.38	N
170	N	N/A	N/A	N/A	3.5	1.6	2.9	2.7	0.97	N
171	N	N/A	N/A	N/A	1.2	2.3	2.1	1.9	0.59	N
172	N	N/A	N/A	N/A	0.9	0.8	1.4	1.0	0.32	N
173	N	N/A	37.23	37.23	0.9	1.3	1.8	1.3	0.45	N
174	N	N/A	N/A	N/A	1.8	0.9	2.1	1.6	0.62	N
175	N	35.22	N/A	35.22	0.8	0.9	1.6	1.1	0.44	N
176	N	32.41	N/A	32.41	1.9	1.5	1.3	1.6	0.31	N
177	N	N/A	32.10	32.10	1.6	2.3	1.8	1.9	0.36	N
178	N	N/A	N/A	N/A	2.1	2.3	1.6	2.0	0.36	N
179	N	37.61	N/A	37.61	1.8	2.1	1.1	1.7	0.51	N
180	N	35.17	N/A	35.17	1.8	1.6	1.7	1.7	0.10	N
181	N	32.92	N/A	32.92	1.0	1.9	1.5	1.5	0.45	N
182	P	24.12	24.10	24.11	11.8	13.5	12.1	12.5	0.91	P

Table S5 Clinical sample testing results by HPV16 qPCR and LAMP-Cas12a/ILR/PGM

Patient #	Clinical HPV16 status	Self conducted HPV16 qPCR (Cq value)			HPV16 LAMP-Cas12a/ILR/PGM					
		Test 1	Test 2	Average	PGM reads (n=3)			Average	STDEV	LAMP-Cas12a/ILR/PGM positive or not
95	N	N/A	N/A	N/A	1.2	0.8	1.5	1.17	0.35	N
96	N	39.93	N/A	39.93	1.3	1.9	0.9	1.37	0.50	N
97	N	N/A	N/A	N/A	1.7	1	1.5	1.40	0.36	N
98	N	N/A	N/A	N/A	1.5	2.1	2.2	1.93	0.38	N
99	N	N/A	N/A	N/A	1.1	0.7	1.5	1.10	0.40	N
100	N	36.16	N/A	36.16	1.8	1.2	0.8	1.27	0.50	N
101	N	N/A	N/A	N/A	1.9	2	1.6	1.83	0.21	N
102	N	N/A	N/A	N/A	0.9	1.3	1.5	1.23	0.31	N
103	P	27.93	28.54	28.24	12.4	12.2	13.1	12.57	0.47	P
104	P	18.81	18.58	18.70	14.3	15.7	16.5	15.50	1.11	P
105	N	32.25	36.80	34.53	1.5	1	1.7	1.40	0.36	N
106	N	32.94	N/A	32.94	1.3	2.2	0.8	1.43	0.71	N
107	N	32.67	30.36	31.52	1.2	0.9	1.1	1.07	0.15	N
108	P	30.14	29.86	30.00	9.6	9.3	8.5	9.13	0.57	P
109	N	N/A	N/A	N/A	0.7	1.8	1.6	1.37	0.59	N
110	N	N/A	N/A	N/A	2.1	2	1.8	1.97	0.15	N
111	N	N/A	38.03	38.03	1.5	1.5	1.3	1.43	0.12	N
112	N	N/A	N/A	N/A	1.7	1.3	0.8	1.27	0.45	N
113	N	32.53	30.81	31.67	1.6	1.2	1.9	1.57	0.35	N
114	N	N/A	N/A	N/A	1	0.9	1.6	1.17	0.38	N
115	N	29.71	N/A	N/A	1.3	1.7	0.9	1.30	0.40	N
116	N	N/A	37.81	37.81	2.2	1.1	1.8	1.70	0.56	N
117	N	38.14	N/A	38.14	1.7	2.5	1.5	1.90	0.53	N
118	N	N/A	39.27	39.27	1.9	0.8	1.3	1.33	0.55	N
119	N	N/A	N/A	N/A	0.9	1.5	2	1.47	0.55	N
120	N	N/A	N/A	N/A	2.2	1.6	1.5	1.77	0.38	N
121	N	37.19	N/A	37.19	2.1	1.1	0.7	1.30	0.72	N
122	N	N/A	N/A	N/A	1.4	1.8	1.2	1.47	0.31	N
123	N	N/A	N/A	N/A	2.2	1.7	1	1.63	0.60	N
124	N	N/A	N/A	N/A	1.5	2	0.9	1.47	0.55	N
125	N	N/A	N/A	N/A	0.8	1.3	1.9	1.33	0.55	N
126	N	N/A	N/A	N/A	0.7	1.8	2	1.50	0.70	N
127	P	29.32	29.31	29.32	10.5	11.2	9.9	10.53	0.65	P
128	N	N/A	N/A	N/A	1.8	2.5	1.6	1.97	0.47	N
129	N	35.91	37.06	36.49	1.7	1	2.1	1.60	0.56	N
130	N	N/A	N/A	N/A	0.9	1.3	1.5	1.23	0.31	N
131	N	N/A	N/A	N/A	1.1	2.2	0.9	1.40	0.70	N
132	N	N/A	N/A	N/A	1.2	2	0.8	1.33	0.61	N
133	N	N/A	N/A	N/A	1.9	1.6	1.8	1.77	0.15	N
134	P	30.05	29.62	29.84	8.4	8.2	9.9	8.83	0.93	P
135	P	27.34	26.77	27.06	8.1	9.4	9.7	9.07	0.85	P
136	N	N/A	N/A	N/A	2.1	1.7	1.3	1.70	0.40	N
137	P	22.25	22.02	22.14	11.9	11.6	12.3	11.93	0.35	P
138	N	N/A	N/A	N/A	1	1.5	1.2	1.23	0.25	N
139	P	21.41	21.09	21.25	12.9	14.8	13.4	13.70	0.98	P
140	N	N/A	N/A	N/A	1.9	0.8	1.1	1.27	0.57	N
141	N	N/A	N/A	N/A	0.9	2.2	1.8	1.63	0.67	N
142	P	27.32	26.02	26.67	12	12.9	13.8	12.90	0.90	P
143	N	N/A	N/A	N/A	1.6	1.9	1	1.50	0.46	N
144	N	39.55	N/A	39.55	2.2	2.5	2	2.23	0.25	N
145	N	N/A	N/A	N/A	1.7	1.2	2.1	1.67	0.45	N
146	N	36.22	35.11	35.67	0.7	0.8	1.9	1.13	0.67	N
147	N	N/A	N/A	N/A	1.3	1.5	0.9	1.23	0.31	N
148	N	N/A	N/A	N/A	1.2	1.8	1.1	1.37	0.38	N
149	P	29.43	29.99	29.71	11.2	12.2	10.6	11.33	0.81	P
150	P	26.12	27.46	26.79	10.8	10.1	10.3	10.40	0.36	P
151	N	N/A	N/A	N/A	1.5	1	1.7	1.40	0.36	N
152	P	24.95	25.32	25.14	11.3	11	12.8	11.70	0.96	P
153	N	N/A	N/A	N/A	0.9	2.1	1.2	1.40	0.62	N
154	P	29.58	30.16	29.87	9.3	10.2	11.4	10.30	1.05	P
155	N	N/A	N/A	N/A	0.8	1.8	1.6	1.40	0.53	N
156	N	N/A	N/A	N/A	1.9	2	1.3	1.73	0.38	N
157	N	39.20	N/A	39.20	1.1	2.1	1.5	1.57	0.50	N
158	N	34.44	N/A	34.44	1	2.2	0.7	1.30	0.79	N
159	N	N/A	N/A	N/A	1.2	2.4	2.1	1.90	0.62	N
160	N	N/A	N/A	N/A	1.6	1.3	0.9	1.27	0.35	N
161	N	N/A	N/A	N/A	1.7	0.8	1.8	1.43	0.55	N
162	N	N/A	N/A	N/A	2.1	1.4	1.9	1.80	0.36	N
163	N	N/A	N/A	N/A	0.8	1.5	2	1.43	0.60	N
164	N	N/A	N/A	N/A	1	1.1	1.8	1.30	0.44	N
165	N	N/A	N/A	N/A	0.7	1.4	1.2	1.10	0.36	N
166	N	N/A	N/A	N/A	1.3	0.9	1.9	1.37	0.50	N
167	N	N/A	N/A	N/A	0.8	1.8	1.7	1.43	0.55	N
168	N	N/A	N/A	N/A	1.9	2.4	1.6	1.97	0.40	N
169	N	N/A	N/A	N/A	2.2	1.9	2.1	2.07	0.15	N
170	N	N/A	N/A	N/A	1.2	1.5	1	1.23	0.25	N

171	N	N/A	N/A	N/A	1.1	2.5	0.9	1.50	0.87	N
172	N	N/A	N/A	N/A	1.3	0.8	0.7	0.93	0.32	N
173	N	N/A	N/A	N/A	1.8	2	1.2	1.67	0.42	N
174	N	39.07	N/A	39.07	1.6	1.7	1.2	1.50	0.26	N
175	N	35.92	N/A	35.92	2.1	1.9	1.5	1.83	0.31	N
176	N	35.24	N/A	35.24	1	0.9	2.2	1.37	0.72	N
177	N	N/A	39.30	39.30	0.8	0.7	1.6	1.03	0.49	N
178	P	29.46	29.21	29.33	10.4	11.7	9.9	10.67	0.93	P
179	N	N/A	N/A	N/A	1.2	1.9	1.1	1.40	0.44	N
180	N	39.03	N/A	39.03	1.8	1	1.3	1.37	0.40	N
181	N	38.48	N/A	38.48	2.1	1.9	1.7	1.90	0.20	N
182	N	36.50	N/A	36.50	0.8	1.5	0.9	1.07	0.38	N

Table S6. Clinical sample testing results by HPV52 qPCR and LAMP-Cas12a/ILR/PGM

Patient #	Clinical HPV52 status	Self conducted HPV52 qPCR (Cq value)			HPV52 LAMP-Cas12a/ILR/PGM					
		Test 1	Test 2	Average	PGM reads (n=3)			Average	STDEV	LAMP-Cas12a/ILR/PGM positive or not
95	N	33.05	31.82	32.44	0.9	0.7	1.2	0.93	0.25	N
96	N	32.55	33.10	32.83	1.1	1.6	0.6	1.10	0.50	N
97	N	33.54	31.42	32.48	0.7	1	1.4	1.03	0.35	N
98	N	30.93	31.45	31.19	1.5	2.2	1.9	1.87	0.35	N
99	N	32.67	33.70	33.19	2	1.8	0.8	1.53	0.64	N
100	P	30.01	29.34	29.68	6.8	7.2	7.3	7.10	0.26	P
101	N	31.37	31.60	31.49	1.7	2.1	1.3	1.70	0.40	N
102	N	33.30	33.19	33.25	1.2	1.5	1.6	1.43	0.21	N
103	P	23.17	22.80	22.99	9.9	8.4	10.1	9.47	0.93	P
104	N	32.68	32.28	32.48	2.3	2.4	1.1	1.93	0.72	N
105	N	36.65	36.08	36.37	1.9	1.4	0.9	1.40	0.50	N
106	N	31.87	33.45	32.66	0.6	1	0.7	0.77	0.21	N
107	N	31.99	31.74	31.87	1.9	2.3	1.2	1.80	0.56	N
108	N	31.61	31.08	31.35	2.5	1.7	1.6	1.93	0.49	N
109	N	31.37	31.03	31.20	2.2	0.8	2	1.67	0.76	N
110	N	29.76	31.00	30.38	1.1	1.5	1.8	1.47	0.35	N
111	N	33.22	31.57	32.40	2.4	1.1	1.6	1.70	0.66	N
112	N	32.35	31.89	32.12	1.4	1.9	1.4	1.57	0.29	N
113	P	29.88	29.21	29.55	7.2	8.1	10.7	8.67	1.82	P
114	N	31.63	31.97	31.80	2.1	1.9	1.3	1.77	0.42	N
115	N	32.08	31.63	31.86	1.9	0.7	0.6	1.07	0.72	N
116	N	32.68	33.30	32.99	0.7	2.6	2.2	1.83	1.00	N
117	N	32.14	31.97	32.06	2.5	1.7	1.5	1.90	0.53	N
118	N	34.56	35.25	34.91	2	0.8	1	1.27	0.64	N
119	N	32.46	34.91	33.69	1.1	1.1	2.3	1.50	0.69	N
120	N	31.88	32.65	32.27	1.4	1.6	0.9	1.30	0.36	N
121	N	32.08	31.43	31.76	1.3	1.2	1.8	1.43	0.32	N
122	N	33.75	32.41	33.08	1.5	2.1	1.7	1.77	0.31	N
123	N	32.53	31.17	31.85	1.9	0.6	0.7	1.07	0.72	N
124	N	31.04	30.11	30.58	1.2	2.2	0.9	1.43	0.68	N
125	N	30.95	31.21	31.08	2.4	1	1.4	1.60	0.72	N
126	N	30.85	30.82	30.84	1.1	2.3	2	1.80	0.62	N
127	N	31.55	30.89	31.22	0.8	1.5	0.8	1.03	0.40	N
128	N	N/A	39.83	39.83	1.6	2.6	1.4	1.87	0.64	N
129	N	29.76	30.46	30.11	0.7	2.5	2.2	1.80	0.96	N
130	N	31.52	32.44	31.98	1.2	1.9	1.3	1.47	0.38	N
131	P	24.08	24.15	24.12	10.2	9.8	11.4	10.47	0.83	P
132	P	20.64	20.29	20.47	10.8	12.3	12.8	11.97	1.04	P
133	N	30.11	30.41	30.26	2.3	1.7	1.2	1.73	0.55	N
134	P	23.89	23.45	23.67	9.7	11.3	10	10.33	0.85	P
135	N	32.26	33.04	32.65	2.4	1.5	1.1	1.67	0.67	N
136	N	31.24	30.36	30.80	0.9	2.2	0.8	1.30	0.78	N
137	N	33.44	35.43	34.44	2.1	1.4	0.6	1.37	0.75	N
138	N	32.05	32.02	32.04	1.3	1.8	1.1	1.40	0.36	N
139	N	30.98	30.70	30.84	1.9	0.7	1.6	1.40	0.62	N
140	N	N/A	34.35	34.35	1.7	1	1.2	1.30	0.36	N
141	P	30.79	28.93	29.86	8.3	6.9	7.6	7.60	0.70	P
142	N	34.22	33.57	33.90	2.5	1.5	2	2.00	0.50	N
143	P	23.07	22.70	22.89	13.2	11.5	11.3	12.00	1.04	P
144	P	20.50	20.60	20.55	12.8	10.3	13.1	12.07	1.54	P
145	P	20.99	21.07	21.03	10.9	10.4	12.5	11.27	1.10	P
146	P	15.10	15.76	15.43	12.8	11	12.2	12.00	0.92	P
147	N	31.55	30.35	30.95	1.1	1.9	1.4	1.47	0.40	N
148	N	31.38	30.12	30.75	1	0.6	0.8	0.80	0.20	N
149	N	31.88	31.18	31.53	1.8	1.3	1.5	1.53	0.25	N
150	N	30.59	31.12	30.86	1.2	0.7	0.9	0.93	0.25	N
151	P	24.56	24.80	24.68	10.9	11.4	11.8	11.37	0.45	P
152	N	32.42	30.96	31.69	1.4	2.6	2.2	2.07	0.61	N
153	P	29.48	30.52	30.00	8.6	7.4	7.2	7.73	0.76	P
154	N	31.56	31.06	31.31	2.1	1.5	0.7	1.43	0.70	N
155	N	32.46	34.83	33.65	0.8	2	1.3	1.37	0.60	N
156	P	26.06	26.15	26.11	9.8	9.5	9.5	9.60	0.17	P
157	N	32.40	31.20	31.80	2.3	1.9	1.6	1.93	0.35	N
158	P	30.39	29.29	29.84	6.8	5.9	6.1	6.27	0.47	P
159	N	31.36	31.16	31.26	2.5	1.7	2.4	2.20	0.44	N
160	N	32.33	32.59	32.46	2.2	1.1	1.4	1.57	0.57	N
161	N	33.70	32.15	32.93	0.7	1	1.8	1.17	0.57	N
162	N	32.41	35.10	33.75	1.2	1.4	0.9	1.17	0.25	N
163	N	36.01	36.18	36.09	2.6	2.1	1.5	2.07	0.55	N
164	N	33.19	32.62	32.91	0.8	1.3	0.6	0.90	0.36	N
165	N	33.02	33.39	33.20	1.5	0.8	2.4	1.57	0.80	N
166	N	33.10	32.51	32.81	1.9	1.4	1.7	1.67	0.25	N
167	N	34.07	32.78	33.42	1.1	2.4	1.2	1.57	0.72	N
168	N	33.17	32.93	33.05	0.9	2	1.1	1.33	0.59	N
169	N	34.19	33.77	33.98	1.6	1.8	2.2	1.87	0.31	N
170	N	33.26	32.85	33.06	2.1	0.7	1.5	1.43	0.70	N
171	N	34.50	33.55	34.02	1.4	1.8	0.7	1.30	0.56	N
172	N	34.37	33.89	34.13	1.3	2.6	1	1.63	0.85	N
173	N	32.97	32.71	32.84	2.4	2.2	1.1	1.90	0.70	N

174	N	34.18	32.88	33.53	0.8	2.5	2	1.77	0.87	N
175	N	32.92	35.47	34.20	1.9	0.6	1.6	1.37	0.68	N
176	N	33.35	33.53	33.44	2.3	2.1	1.4	1.93	0.47	N
177	N	33.08	33.42	33.25	2	1.2	1.8	1.67	0.42	N
178	N	34.10	32.10	33.10	0.7	1.5	1.1	1.10	0.40	N
179	N	33.49	33.67	33.58	0.9	1.3	1.6	1.27	0.35	N
180	N	34.30	34.27	34.29	1.7	1.4	0.8	1.30	0.46	N
181	P	16.83	17.05	16.94	13.9	14.5	12.4	13.60	1.08	P
182	N	32.44	32.77	32.61	1	0.6	1.2	0.93	0.31	N

Table S7. Preceding HPV detection development

Category	System	Target	Readout	Pre-amplification	POCT potential	LOD	Clinical samples	Sensitivity and specificity	Reference PMID or DOI
CRISPR-Cas-Mediated	Cas12a/ILR/PGM	HPV18; HPV16; HPV52	PGM	LAMP	YES	~7 HPV18 positive cells per reaction	182, 88 and 88 cervical cellular samples	95.8% and 100; 100% and 100%; 100% and 100%	This work
	Cas12a	13 types of high-risk HPVs	Fluorescence	RPA	NA	500 copies per reaction	3 samples each type	100% and 100%	34140632
	Cas12a	HPV-16; HPV-18	Fluorescence	RPA	NA	1 aM	25 patient samples each type	100% and 100%; 75% and 100%	29449511
	Cas12a	HPV-16	Electrochemical	NA	YES	50 pM	NA	NA	31568601
	Cas12a	HPV-16; HPV-18	SERS	NA	NA	1 aM	NA	NA	34369760
	Cas12a	HPV-16; HPV-18	Fluorescence	NA	NA	38.7 fM	15 patients	100% and 100%; 100% and 100%	10.1016/j.snb.2023.134813
	Cas12a	HPV-16; HPV-18	Fluorescence	NA	YES	5 fM	NA	NA	10.1002/aic.17365
	Cas12a	HPV-16	Electrochemiluminescence (ECL)	NA	NA	8.86 fM	NA	NA	37402133
Others	Electrochemical Immunosensor (GCE-OLC-PAN)	HPV-16 L1 (antige	Electrochemcal	NA	NA	0.61 fg/mL (10.89 m aM)	NA	NA	37384904
	Microfluidic portable all-in-one device (PAD)	HPV-16; HPV-18	colorimetric	LAMP	Yes	1 copy/μl	206 clinical samples	92.1% and 99.0%	38150799
	SlipChip-based Integrated Point-of-Care (SIPOC) system	HPV-16; HPV-18	Fluorescence	qPCR	NA	200 copies/mL	130 participants	96.2% and 100%; 90.0% and 100%	39320328
	Self-digitization (SD) microfluidic chip	HPV-18	Fluorescence	digital LAMP	NA	NA	NA	NA	30734822
	distance microfluidic paper-based analytical devices (dμPAD)	E7 mRNA	Fluorescence	RCA	Yes	10 fM	40 samples	87.5% and 93.5%	38912660
	LFA	14 high-risk HPV type	colorimetric	RPA	Yes	1~10 copies of HPV DNA per reaction	198 samples	96% and 83%	37606488
	Oligonucleotide capped nanoporous anodic alumina films sensor	14 high-risk HPV type	Fluorescence	NA	NA	NA	43 samples	100% and 93-100%	37285852
	Artificial chaperone-enhanced MNAzyme (ACEzyme) system	HPV-16	Electrochemical	NA	NA	0.88 pM	NA	NA	35690559
	ECL sensing system	HPV 16	Electrochemiluminescence (ECL) sensor	NA	NA	0.03 nmol L−1.	NA	NA	32339153
